# Tracking Persistent Symptoms in Scotland (TraPSS): a longitudinal prospective cohort study of COVID-19 recovery after mild acute infection

**DOI:** 10.1136/bmjopen-2024-086646

**Published:** 2025-01-15

**Authors:** Nicholas F Sculthorpe, Marie McLaughlin, Luke Cerexhe, Eilidh Macdonald, Antonio Dello Iacono, Nilihan E M Sanal-Hayes, Joanne Ingram, Rachel Meach, David Carless, Jane Ormerod, Lawrence D Hayes

**Affiliations:** 1 Sport and Physical Activity Research Institute, University of the West of Scotland, Glasgow, UK; 2 Institute for Sport Physical Education and Health Sciences, University of Edinburgh, Edinburgh, UK; 3 School of Health and Society, University of Salford, Salford, UK; 4 Durham University, Durham, UK; 5 Long COVID Scotland, Aberdeen, UK; 6 Lancaster University Medical School, Lancaster University, Lancaster, Lancashire, UK

**Keywords:** COVID-19, Epidemiology, Post-Acute COVID-19 Syndrome

## Abstract

**Abstract:**

**Background:**

COVID-19 disease results in disparate responses between individuals and has led to the emergence of long coronavirus disease (Long-COVID), characterised by persistent and cyclical symptomology. To understand the complexity of Long-COVID, the importance of symptom surveillance and prospective longitudinal studies is evident.

**Methods:**

A 9-month longitudinal prospective cohort study was conducted within Scotland (n=287), using a mobile app to determine the proportion of recovered individuals and those with persistent symptoms and common symptoms, and associations with gender and age.

**Results:**

3.1% of participants experienced symptoms at month 9, meeting the criteria for Long-COVID, as defined by the National Institute for Health and Care Excellence terminology. The random effects model revealed a significant time (month) effect for infection recovery (p<0.001, estimate=0.07). Fatigue, cough and muscle pain were the most common symptoms at baseline, with fatigue persisting the longest, while symptoms like cough improved rapidly. Older age increased the likelihood of reporting pain (p=0.028, estimate=0.07) and cognitive impairment (p<0.001, estimate=0.93). Female gender increased the likelihood of headaches (p=0.024, estimate=0.53) and post-exertional malaise (PEM) frequency (p=0.05, estimate=137.68), and increased time x gender effect for PEM frequency (p=0.033, estimate=18.96).

**Conclusions:**

The majority of people fully recover from acute COVID-19, although often slowly. Age and gender play a role in symptom burden and recovery rates, emphasising the need for tailored approaches to Long-COVID management. Further analysis is required to determine the characteristics of the individuals still reporting ongoing symptoms months after initial infection to identify risk factors and potential predictors for the development of Long-COVID.

**STRENGTHS AND LIMITATIONS OF THIS STUDY**

- This study examined symptomology following an acute COVID-19 infection for 9 months, specifically in Scotland.
- The primary strength was the use of prospective, rather than retrospective, symptom tracking.
- A secondary strength was our utility of mobile health for inclusivity and low participant burden.
- A limitation is that at the time of study commencement, definitions around acute, post-acute and persistent symptoms were still being refined by the National Institute for Health and Care Excellence.
- A second limitation is the moderate sample size.

## Introduction

 Since its emergence in late 2019, the coronavirus disease, severe acute respiratory syndrome coronavirus 2 (SARS-CoV-2), has rapidly spread across the globe, with >760 million confirmed infections.^1^ Although there is considerable disagreement in the exact number of deaths from coronavirus disease (COVID)-19,^2 3^ some reports have estimated the number as >6.9 million worldwide.^1^ Acute responses to infection vary widely, ranging from individuals who may be asymptomatic to those who experience severe respiratory distress or other organ damage.^4^ The UK’s National Institute for Health and Care Excellence (NICE) has categorised the duration of these COVID-19 symptoms into three distinct phases: acute (<4 weeks), subacute (4–12 weeks) and chronic (>12 weeks), with the latter two intervals collectively recognised as long coronavirus disease (‘Long-COVID’). Prevalence estimates for Long-COVID vary, ranging from 13% in select community-based cohorts with laboratory-confirmed COVID-19 to upwards of 71% in hospitalised patients.^5^,^7^ It is worth noting however that even before COVID-19, nearly one-fifth of patients discharged from a hospital develop an acute medical problem within the subsequent 30 days that cause another hospitalisation,^8^ which may account for the range in prevalence rates between hospitalised patients and non-hospitalised patients.

Our recent scoping review highlighted more than 100 symptoms of Long-COVID.^9^ However, Davis and colleagues^10^ estimated 203 symptoms across 10 organ systems in an online survey of people with suspected and confirmed COVID-19, from 56 countries with a symptom duration of >28 days. This is conflicting with a living systematic review by Michelen *et al*^11^ who reported over 60 physical and psychological symptoms. It is possible that the study by Michelen *et al*^11^ may have excluded many studies as an inclusion criterion for individual studies was ‘at least 100 people with confirmed or clinically suspected COVID-19 at 12 weeks or more post onset’. In the early phase of the pandemic, most studies concerning Long-COVID had small sample sizes^9^ so may have not been included in this systematic review. Regardless of the precise number of symptoms, it is evident from the scientific literature and patient support groups that Long-COVID is a complex condition with heterogeneous symptomology.^12^ This makes the formation of a precise case definition or risk evaluation challenging, evidenced by the duration which the Long-COVID Core Outcome Set (LC-COS) took to produce.^13^ That being said, now the literature base has increased, and it is apparent that fatigue is possibly the most common symptom reported by people with Long-COVID,^14 15^ and qualitative studies have detailed how debilitating this is for people.^16 17^

Much early work considering Long-COVID was either retrospective or cross-sectional in nature or conducted in specialised units, providing little information about the natural history of the progression from acute infection to either recovery or Long-COVID.^9 10^ More recently, prospective studies emerged which provide some insight, although data were often limited. For example, Bai *et al*^18^ undertook a prospective study of patients recovering from a COVID-19 infection over a 3-month period. They reported that being female, active smoking and increased age were risk factors for progression to Long-COVID. They also reported nearly 70% of patients received a diagnosis of Long-COVID. However, their high prevalence rate was likely influenced by their data coming from a specific post-COVID outpatient service, with most patients having been both hospitalised and intubated during their acute infection. Consequently, it is unclear how well this kind of prospective data reflects the natural progression of the condition in many people who remained community-dwelling and were never hospitalised during the acute phase.

Other prospective studies have reported similar prevalence rates over 6^19^ or 12 months^20^ using larger, more representative samples, and other meta-analyses and data pooling resulted in smaller estimates.^21 22^ While these studies provide valuable estimates of prevalence, they provide limited information regarding the natural history of COVID-19 infection. Indeed, few studies have examined the longitudinal evolution of symptoms starting from the point of acute infection. Most have only a single follow-up, making it difficult to assess the longitudinal changes in symptom load. Finally, most studies only undertake symptom counting and fail to include broader assessments of patient-reported outcome measures such as the LC-COS using validated instruments at regular time points.^23^ This has led to calls for prospective, robust, standardised, controlled studies to characterise Long-COVID in different at-risk populations and settings.^11^

A clearer picture of the natural history and long-term sequelae after COVID-19 infection is needed to inform management and treatment. Therefore, the aim of this project was to track symptoms of individuals following a COVID-19 infection for 9 months using a specially designed mobile health app to determine symptomology changes over time and to undertake regular assessments with validated instruments. Our objectives were (1) to evaluate the natural history of symptoms post-infection in Scotland, (2) to detect the proportion of people who have persistent symptoms, (3) to identify the most common symptoms associated with COVID-19 recovery and their relative frequencies and (4) to identify associations of gender and age with symptom recovery.

## Methods

### Study design

**Figure 1:**
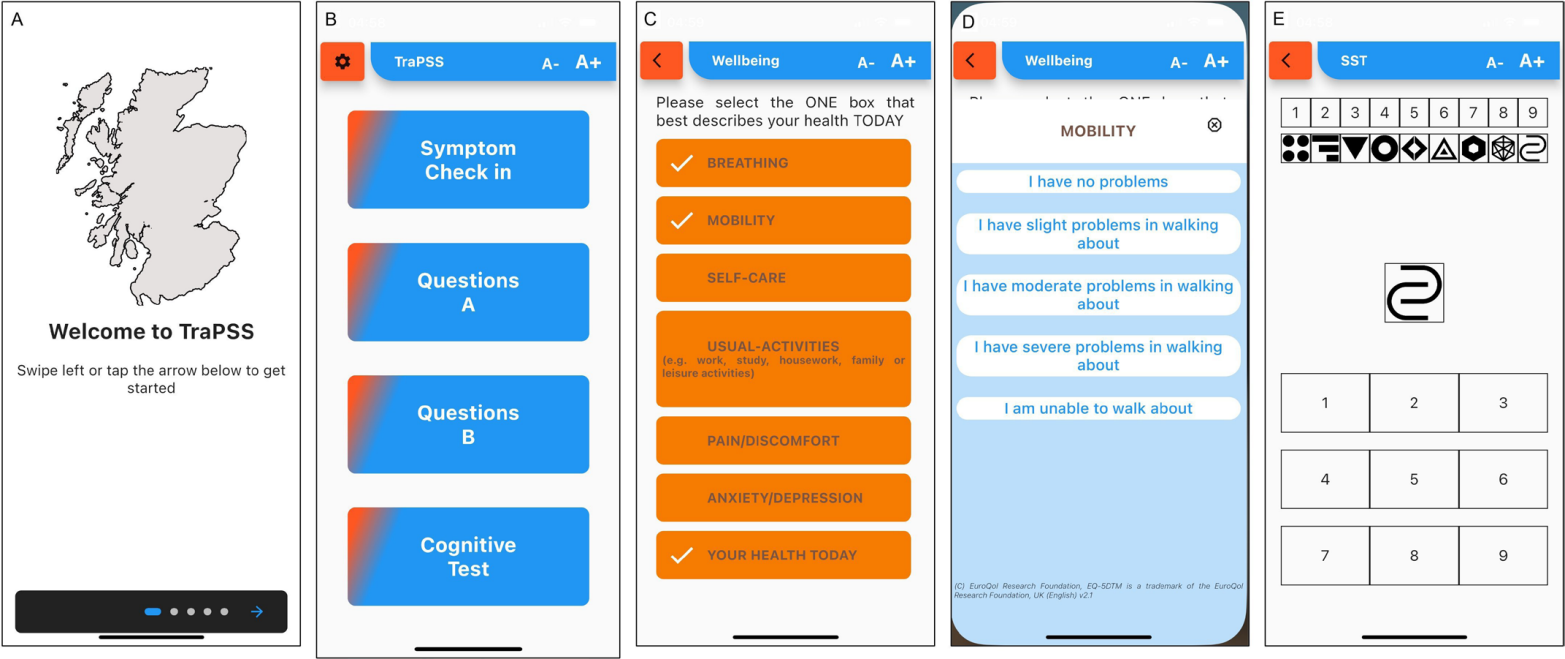
Screen shots of the bespoke Tracking Persistent Symptoms in Scotland (TraPSS) app. (A) Onboarding screen, (B) main home screen, (C) symptom check-in screen, (D) Visual Analogue Scale (VAS) for current health item from the EuroQol-5 Dimension (EQ5D), (E) instructions for the cognitive function test.

A 9-month longitudinal prospective tracking study was conducted within Scotland using a bespoke mobile app—‘Tracking Persistent Symptoms in Scotland (TraPSS)’ (figure 1). Once per month, participants were required to ‘check-in’ by completing a set of instruments within the app, which contained questions regarding COVID-19 symptoms, validated questionnaires regarding general health and well-being, and a cognitive function test. Participants were sent a notification reminder to complete the app every 31 days but were able to complete the app as often as they wished. At each check-in, responses took ~20 min to complete.

### Patient and public involvement

Long COVID Scotland were the participant and patient involvement (PPI) partners for the project and became involved prior to the initial proposal. Our PPI lead became part of the project team and liaised with Long COVID Scotland’s members regarding the design, the selection of instruments and useability testing of the mobile tracking app. Our PPI lead advocated for participants during discussions of study management and progression. Following the project’s completion and publication of final results, further feedback will be provided to Long COVID Scotland, who will support further dissemination of the findings through their networks.

### Inclusion criteria

Participants were included in the study if they were adults (≥18 years) living in Scotland and self-report a positive COVID-19 test (any type of assay) within the previous 10 weeks. Respondents were excluded for insufficient English language for messaging to be effective; no mobile device access; impaired cognitive function which compromised comprehension of study information or messaging; current participation in any COVID-19 intervention and receiving therapies known to cause symptom exacerbations (eg, chemotherapy).

### Participant recruitment

Participants were recruited via snowball sampling using social media posts on Facebook/Meta and Twitter/X between January 2022 and January 2023. Expressions of interest from participants were met with an invitation to a one-to-one virtual meeting with a member of the research team. Here, the study was fully explained, and the researcher determined participant eligibility to participate. This meeting also gave participants a chance to ask questions or raise concerns before participating.

### Protocol for downloading the app

The research team provided instructions and technical guidance on downloading and completing the TraPSS app on an Android or iOS device. Participants searched for the app on iOS or Android devices using the search term ‘TraPSS’. Once downloaded, participants created an account using a personal email address. Then, the app took participants through a set of onboarding screens designed to explain the features of the app. Completion of onboarding required participants to consent to take part in the study and provide a digital signature to proceed. A screengrab of the consent screen and signature was captured and remotely stored. After completing consent, the app gathered some basic demographic data, including gender, height, body mass, underlying medical conditions and vaccination status.

### App design

At the time of development, the LC-COS had not been finalised. However, the initial Delphi survey had been completed, outlining the relevant key domains and, consequently, instruments included in the app reflected these.^23^ Where we subsequently refer to mapping to LC-COS in this manuscript, we mean we mapped to the LC-COS domains. The team were also mindful to select instruments that were both valid but minimised participant burden. Consequently, the main interface was split into four sections, with each section to be completed at least once per month. Data collected via the app was stored on a GDPR-compliant server, with data accessed and downloaded using an automated Python script. In addition, each day, the server sent reminder notifications to participants who had not yet completed that week’s questions.

The four sections of the main screen comprised a symptom check-in, two sets of validated instruments grouped into ‘set A’ and ‘set B’ for ease of access, and a cognitive test. The symptom check-in included a single-item assessment mapping to LC-COS recovery (adapted from Tong *et al*^24^) and single response items regarding changes in work circumstances (LC-COS work/occupational changes), and the ability to report new COVID-19 infections. For clarity, we considered ‘recovery’ using a single-item question from Tong *et al*^24^ which is on a 5-point Likert scale (not recovered at all, somewhat recovered, about half recovered, mostly recovered and completely recovered). Finally, participants could identify current symptoms from a list of 14 based on a scoping review of symptoms^9^ and report the frequency (days/week) with which they experienced the symptom. If participants had symptoms not on the list, they could speak or type additional symptoms into the app.

Question set A included assessments of the quality of life using the 12-Item Short Form Health Survey (SF-12^25^; LC-COS physical functioning), the presence of post-exertional malaise (PEM) using the modified PEM Questionnaire^26^ (LC-COS post-exertion symptoms) and the Edinburgh Neurological Questionnaire to assess for the presence of other neurological symptoms^27^ (LC-COS nervous system functioning).

Question set B included the Medical Research Council (MRC) dyspnoea scale^28^ assessing breathlessness/dyspnoea against the ability to carry out activities of daily living (LC-COS respiratory functioning); the European Quality of Life-5 Domains (EQ5D)^29^ to assess anxiety/depression, impairments in mobility, pain, impairments in self-care and impairments in activity (LC-COS mental functioning); and the Patient Health Questionnaire-4^30^ (PhQ4) to assess anxiety and depression where a score ≥3 for the first two questions suggests anxiety and a score ≥3 for the last two questions suggests depression (LC-COS mental functioning). Set B also included a Visual Analogue Scale (VAS) to grade pain on a scale of 0 (no pain at all) to 100 (worst pain imaginable), and self-management was assessed using the self-efficacy for long-term conditions^31^ which graded self-efficacy on a scale of 0 to 100 for disease-specific self-confidence.

Finally, the fourth section contained a Single Digit Modalities Test^32^ (LC-COS cognitive functioning). Shapes appeared on the screen, and participants attempted to identify which number (0–9) according to the grid at the top of the screen the shape corresponded to. The number of correctly identified shapes and time to completion were analysed.

### Data handling

Data collected via the mobile app was stored as anonymised files on a cloud-based protected server to which only the research team had access. Python script was used to download the data from the server into comma-separated values (CSV) files, which were converted into Excel for initial data cleaning.

### Statistical analysis

Descriptive data is presented as mean±SD unless otherwise stated for demographics including gender and age, underlying health condition and vaccination status. To examine the effects of time, age and gender on the construct of recovery, we used the following linear mixed-effects model:

All domains of recovery represented the repeated-measures *outcome* for subject_in_ and served as outcome measures whereas *time* (continuous variable with nine levels (consecutive months)), *gender* (categorical variable with two levels (female and male)) and age (continuous variable) were modelled as predictors and treated as fixed effects alongside their three-way and two-way pairwise interactions. Moreover, random effects were assumed for *participants*, with random slopes per the predictor time introduced in the model as this addition did not result in a convergence error. We assumed data were missing at random and linear mixed-effects models handle missing data without requiring imputation.^33^ Estimated marginal means and 95% CIs were calculated alongside comparisons made using post hoc Holm-Bonferroni adjustments. Visual inspection of residual plots was used to confirm the assumptions of homoscedasticity or normality, which was also assessed through the Shapiro-Wilk test. Moreover, since regression models can be sensitive to multicollinearity, we computed the variance inflation factors for all predictor parameters used in the linear mixed-effects model to inspect the presence of autocorrelation between pairs of predictors. Model residuals were qualitatively examined for structure and heteroscedasticity. We computed 90% CIs of the adjusted effects using the bias-corrected and accelerated bootstrap with 5000 replicates. All statistical analyses were conducted in R language and environment for statistical computing using the *lme4*, *lmerTest*, *emmeans* and *ggeffects* packages while model assumptions were checked using the *performance* package (V.4.0.5; R Core Team, Vienna, Austria). GraphPad Prism 9 was used to create all figures.

$$\begin{array}{l} Outcome_{in}\;=\;\beta_{0}+\beta_{1}\cdot time_{in}+\beta_{2}\cdot gender_{in}\\ +\beta_{3}\cdot age_{in}+\beta_{4}(time_{in}\cdot gender_{in})\\ +\beta_{5}(time_{in}\cdot age_{in})+\beta_{6}(gender_{in}\cdot age_{in})\\ +\beta_{7}(time_{in}\cdot gender_{in}\cdot age_{in})+(1|Subject)+\varepsilon_{i} \end{array}$$

## Results

Using an online form, 471 people expressed interest in taking part in the study. We then contacted these 471 people via email to provide study information and attempt to schedule an online meeting. Of these 471 emails, five people provided an ineligible email address so our email was undelivered. Of the 466 valid emails we sent, 13 people were ineligible (6 people had an acute infection >10 weeks previously, 6 people already had Long-COVID and 1 was <18 years of age). Of the remaining 453 people, 141 did not respond to our email. The remaining 312 either dropped out between our email and the online meeting or did not attend the online meeting. Of the 288 who attended a meeting and were sent the link to enrol, 1 person had an incompatible device, so 287 participants were enrolled. The mean duration from initial infection to enrolment (baseline) was 35±19 days. Participant demographics can be found in online supplemental table 1.

### Infection recovery and change to work

**Figure 2:**
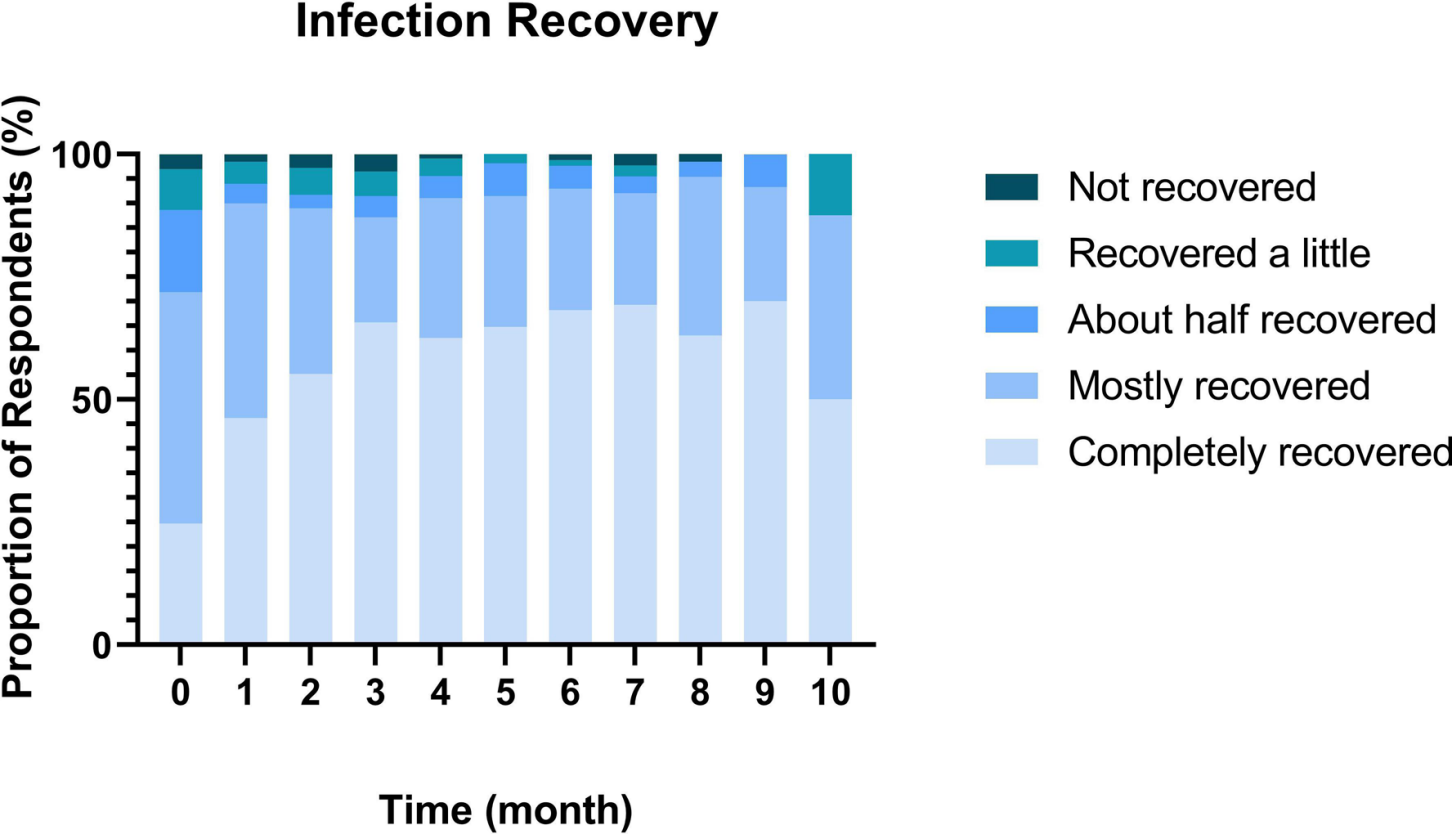
Recovery from initial infection across 9 months, based on single-item assessment (adapted from Tong *et al*^24^).

The proportion of participants reporting ‘completely recovered’ increased from 31.7% at baseline to 96.9% at month 9 (figure 2). The 3.1% not reporting ‘completely recovered’ at month 9 reported being ‘mostly recovered’ (2.4%) or ‘about half recovered’ (0.7%). The random effects model revealed a significant time (month) effect for infection recovery (p<0.001, estimate=0.07). Throughout the study, reporting of new infections was low, with 2% at month 1; 1.7% at month 2, 0.3% at months 3, 4, 6 and 7; and 1% at month 8.

At baseline, 4.4% of participants had decreased work hours, 3.3% had stopped work completely and 7.1% had increased work hours. Results were similar at month 9; 4.3% had decreased work hours, 3.3% had stopped work completely and 11.6% had increased work hours. Reasons why work changed ranged from poor health (92% at baseline to 57% at month 1, 25% at month 3, 33% at month 4, 50% at month 7) to new caring responsibilities (7% at baseline to 14% at month 1, 25% at month 3) to other (0% at baseline to 28.6% at month 1, 50% at month 2, 66.7% at month 3, 100% at months 4–6, 50% at month 7). There was an insufficient number of responses to perform statistical analyses on these.

### Symptom frequency

**Figure 3:**
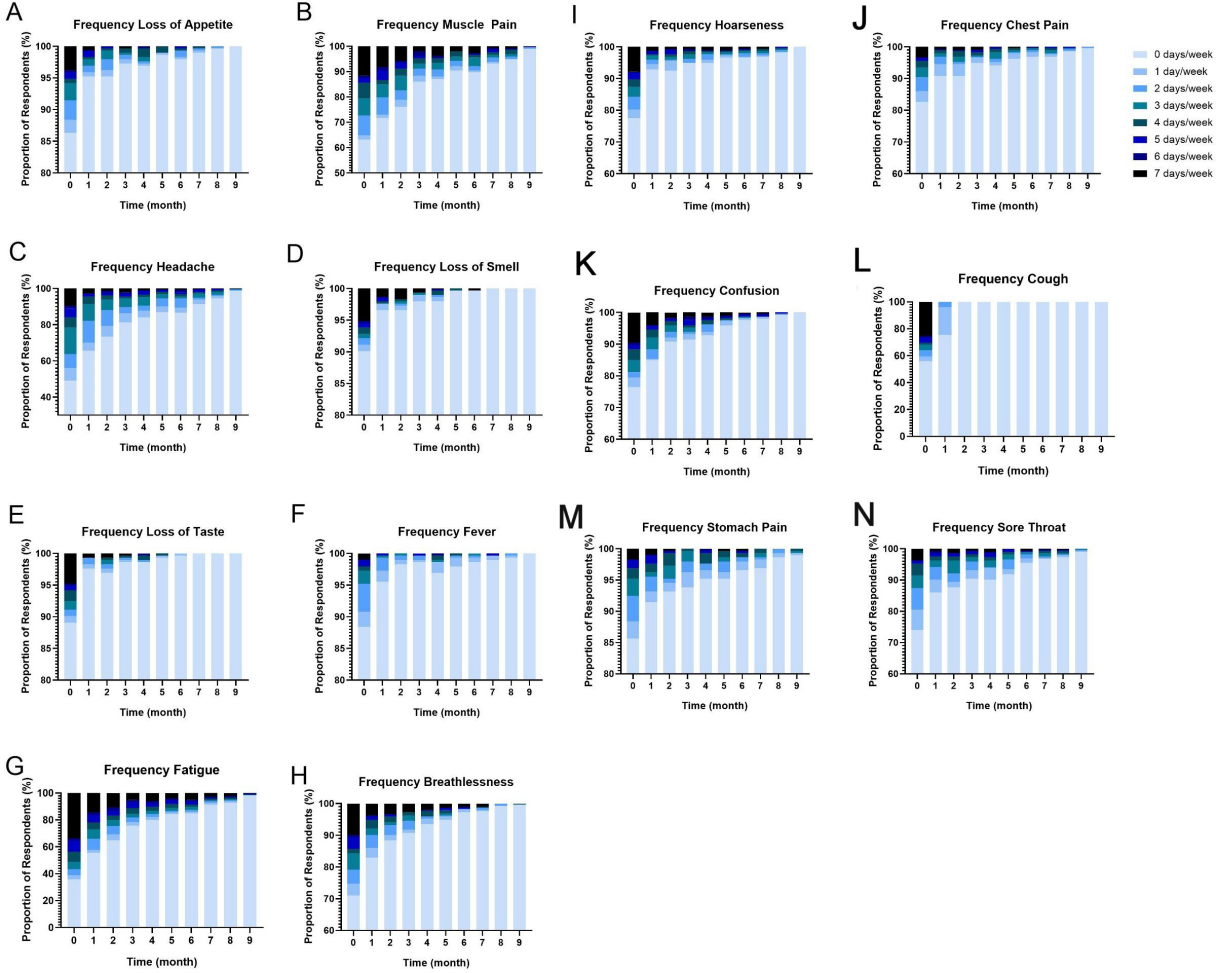
Symptom frequency over time from baseline to month 9, on a scale from 0 to 7 days/week, assessing loss of appetite (**A**), muscle pain (**B**), headache (**C**), loss of smell (**D**), loss of taste (**E**), fever (**F**), fatigue (**G**), breathlessness (**H**), hoarseness (**I**), chest pain (**J**), confusion (**K**), cough (**L**), stomach pain (**M**) and sore throat (**N**).

Fatigue was the most prevalent symptom at baseline, with 64.2% of participants reporting some level of appetite loss (13.7%; figure 3A), muscle pain (36.9%; figure 3B), ‘other symptoms’ include headache (50.9%; figure 3C), loss of smell (9.9%; figure 3D), loss of taste (10.9%; figure 3E), fever (11.6%; figure 3F), fatigue (figure 3G), breathlessness (29.1%; figure 3H), hoarseness (22.5%; figure 3I), chest pain (17.4%; figure 3J), confusion (23.5%; figure 3K), cough (44.0%; figure 3L), stomach pain (14.3%; figure 3M) and sore throat (25.1%; figure 3N).

At baseline, fatigue, cough and muscle pain were the most frequently reported symptoms, with 33.8%, 25.3% and 11.6% reporting an occurrence of 7 days/week, respectively. Fatigue was also the longest-lasting symptom, with 7.2% reporting some level of fatigue in month 8. Muscle pain was also long-lasting, with 5.2% of participants reporting some level of muscle pain at month 8 (decreasing from 36.9% at baseline). Cough was the fastest recovering symptom, with only 14% reporting cough frequency of 1 day/week by month 3.

The mixed-effects models revealed a significant effect of time when controlling for participants’ gender and age for most symptoms including: decreased muscle pain (p=0.004, estimate=−0.17), headache (p<0.001, estimate=−0.18), fatigue (p<0.001, estimate=−0.34), fever (p<0.001, estimate=−0.02), cough (p=0.001, estimate=−0.19), confusion (p<0.001, estimate=−0.11), breathlessness (p<0.001, estimate=−0.12), loss of smell (p<0.001, estimate=−0.05), loss of taste (p<0.001, estimate=−0.04), sore throat (p<0.001, estimate=−0.07), hoarseness (p<0.001, estimate=−0.08), chest pain (p<0.001, estimate=−0.05), stomach pain (p<0.001, estimate=−0.04) and appetite (p<0.001, estimate=−0.04). There was a significant gender (male) effect for decreased headache (p=0.024, estimate=−0.53) and fatigue (p=0.042, estimate=−0.70) and a significant gender (male) × time (month) effect for decreased fatigue only (p=0.020, estimate=0.09). There was no significant effect of age on any symptom (all p>0.05).

### EQ5D

**Figure 4:**
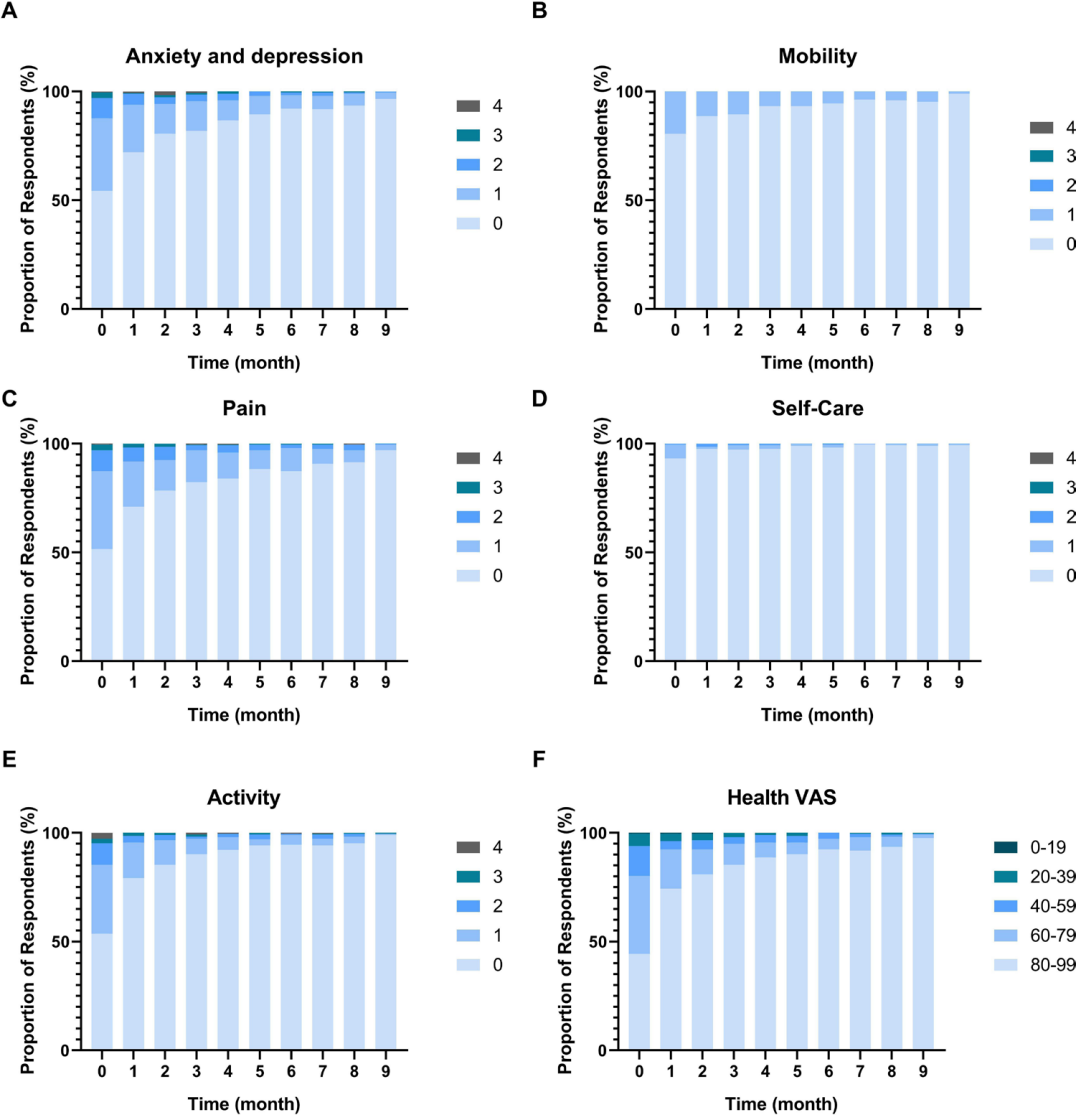
Results from European Quality of Life-5 Domains (EQ5D) health questionnaire over time from baseline to 9 months for anxiety and depression (**A**), impairments in mobility (**B**), pain (**C**), impairments in self-care (**D**), impairments in activity (**E**) and health Visual Analogue Scale (VAS) (**F**). 0–4 indicate the least to most severe responses to the EQ5D questions (ie, 0=‘I have no problems …’, 4=‘I am unable to…’).

From the EQ5D questionnaire, 45.8% of participants reported some level of anxiety and depression at baseline, decreasing to 3.4% at month 9 (figure 4A). Impairments in mobility and self-care are reported by 19.5% and 6.8% of participants at baseline to 1.1% and 0.7% at month 9, respectively (figure 4B,C, respectively). Some level of pain was reported by 48.5% of participants at baseline, decreasing each month to 3.1% at month 9 (figure 4C). Activity levels had decreased in 46.4% of participants at baseline, decreasing to 1.0% at month 9 (figure 4E). 65.6% of participants had reported less than 80% health on VAS at baseline, recovering to 2.4% at month 9 (figure 4F). The random effects model revealed a significant time effect for EQ5D anxiety and depression (p<0.001, estimate=−0.05), impairments in mobility (p<0.001, estimate=−0.02), pain (p<0.001, estimate=−0.05), impairments in self-care (p=0.001, estimate=−0.01), impairments in activity (p<0.001, estimate=−0.05) and VAS health (p<0.001, estimate=1.93).

### Dyspnoea, VAS pain, anxiety/depression and self-efficacy

**Figure 5:**
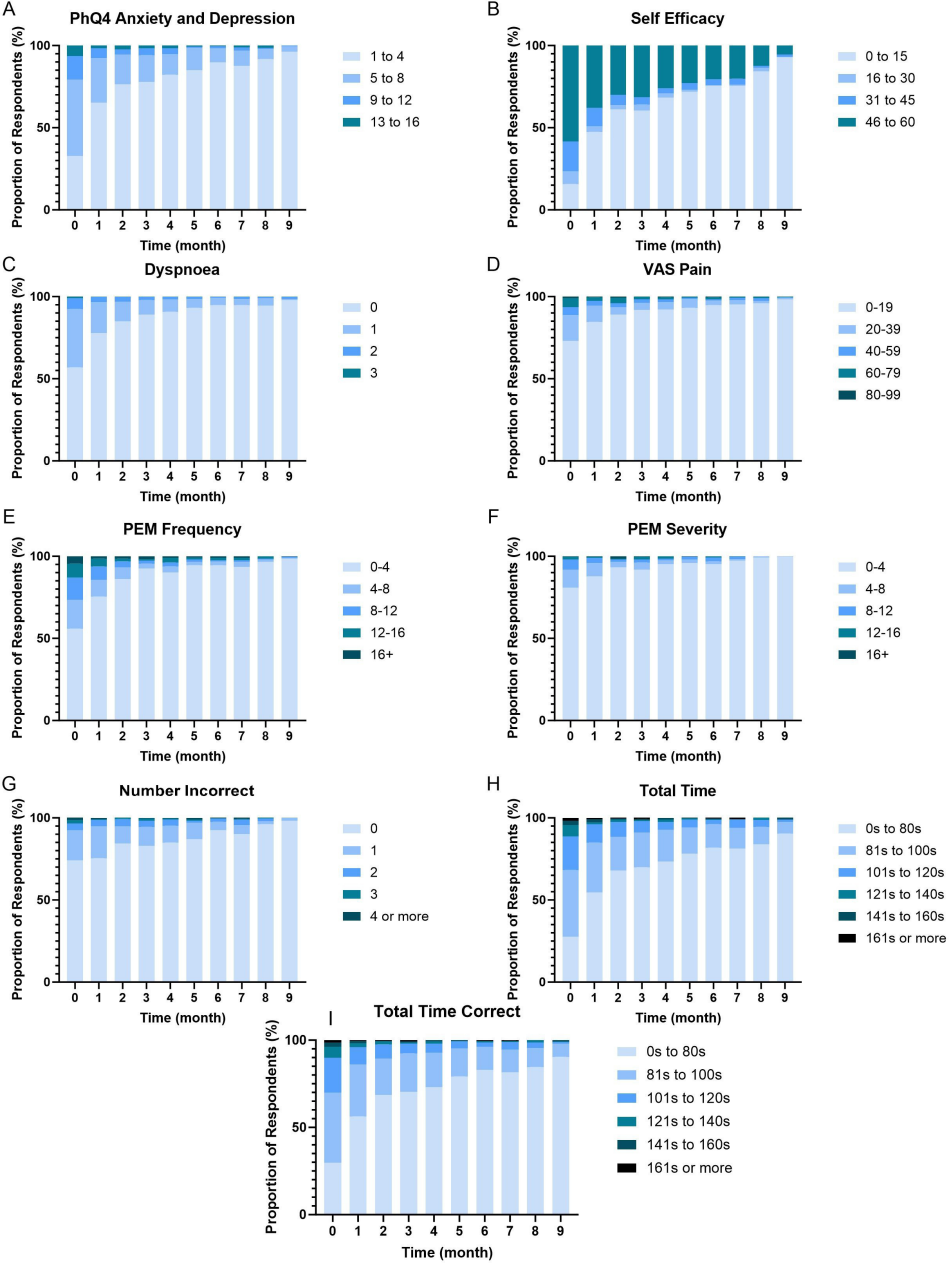
Patient Health Questionnaire-4 (PhQ4) Anxiety and Depression (**A**), Self-Efficacy scores (**B**), Medical Research Council (MRC) Dyspnoea scores (**C**), Visual Analogue Scale Pain (**D**), Post-exertional malaise frequency (**E**) and severity (**F**), Symbol Digit Modalities Test (SDMT): number incorrect (**G**), SDMT: Total time to completion (**H**) and SDMT: total time for correct answers (**I**), from baseline to 9 months.

Anxiety and depression in the PhQ4 were reported by 20.8% of participants at baseline and 0.3% at month 9 (figure 5A). Reduced self-efficacy of condition management was experienced by 84.3% of participants at baseline and 7.2% at month 9 (figure 5B). Dyspnoea was experienced by 44% of participants at baseline, with 2.1% still reporting dyspnoea at month 9 (figure 5C). Pain was reported by 27% of participants at baseline, decreasing to 1.7% at month 9 (figure 5D).

The random effects model revealed a significant time (month) effect for MRC dyspnoea (p<0.001, estimate=−0.04), self-efficacy (p<0.001, estimate=−0.08), VAS pain (p<0.001, estimate=−1.15) and PhQ4 anxiety and depression score (p<0.001, estimate=−1.08). There was a significant effect of age for VAS pain (p=0.028, estimate=0.07) and PhQ4 anxiety and depression score (p<0.001, estimate=−0.88).

### Post-exertional malaise

The frequency (figure 5E) and severity (figure 5F) of PEM decreased from baseline to month 9. At baseline, 44.0% of respondents reported mildly to severely frequent PEM. This decreased to 1.4% at month 9. The severity of PEM decreased from 19.1%, reporting mild to severe fatigue at baseline, to 0.4% at month 9. The random effects model revealed a significant time effect for PEM severity (p<0.001, estimate=−22.64) and frequency (p<0.001, estimate=−39.16). There was a significant gender (male) effect for PEM frequency (p=0.05, estimate=−137.68). There was a significant gender (male) × time (month) effect for PEM frequency (p=0.033, estimate=18.96).

### Cognitive function

The number of incorrectly identified objects on the Symbol Digit Modality test reduced over time from 26.0% getting one or more incorrect at baseline to 1.8% at month 9. Those taking a short time (<80 s) to complete the test at baseline was 27.6% and increased to 90.4% at month 9. Correct answers increased from 29.7% at baseline to 90.4% at month 9.

There was a significant time (month) effect for Symbol Digit Modalities total time (p<0.001, estimate=−1.01), correct time (p<0.001, estimate=−1.08) and number incorrect (p<0.001, estimate=−0.04). There was also a significant effect of age for total time (p<0.001, estimate=0.93) and correct time (p<0.001, estimate=0.88).

## Discussion

This study aimed to (1) evaluate the natural history of symptoms post-infection in Scotland; (2) detect the proportion of people with persistent symptoms, identify common symptoms associated with COVID-19 recovery; and (3) identify associations of gender and age with symptomology recovery. We feel we met these aims, and the main findings were as follows: (1) around a third of people self-reported full recovery within the first month after infection, rising to three-quarters by 3 months, meaning around a quarter of participants met the criteria for Long-COVID set out by NICE (ie, symptoms persisting beyond 12 weeks^34^). (2) Although fatigue was the most frequent and enduring symptom, more than half of people had no symptoms or recovered relatively quickly. For some, however, recovery can be slow, with 1 in 5 still reporting symptoms after 6 months and 1 in 30 at 9 months. (3) Time since infection was the only predictor of recovery for most outcomes, with males recovering more quickly for fatigue and PEM than females. (3) A majority experienced minimal to no symptoms, a subset recovered slowly and a small fraction displayed persistent symptoms throughout the study, which was consistent across domains. Consequently, the prevalence of long-COVID varied substantially (between 40% and 3% depending on the time frame (3 months vs 9 months post-infection) and symptoms being considered). Taken together, these data suggest that recovery from COVID-19 is slower than that from other viral infections and our data highlight the importance of considering duration of infection when assessing Long-COVID prevalence. This has important repercussions for healthcare practitioners when advising patients on recovery from acute infections, but also economic repercussions, given the large amount of people out of work in the UK as a result of long-term illness.^35^,^37^

### Self-reported recovery and symptom load

By 3 months after infection, many reported partial recovery despite also reporting symptoms such as muscle pain and fatigue. This discrepancy could stem from COVID-19’s variable symptom load or individuals feeling mostly recovered except for a few persistent issues. Recovery within 3 months was common and higher than reported elsewhere,^38 39^ potentially due to our sample including only non-hospitalised individuals. Exclusively hospitalised patient studies show lower recovery rates up to a year post-infection.^40 41^ By 9 months, most felt fully recovered, though a small proportion (3.1%) still reported symptoms, aligning with Long-COVID criteria. This is similar to estimates of Long-COVID in Scotland from the Office for National Statistics (ONS),^42^ and if population, it would equate to >1 70 000 people in Scotland with additional healthcare needs. The symptom data support this overall view that for a sizeable minority, recovery from COVID-19 can be slow and aligns with several other studies.^41 43^ Furthermore, while it is lower than some prospective studies,^19 20^ the differences are again likely due to those studies having a mix of hospitalised and non-hospitalised participants.

### Questionnaire responses

A novel aspect of the present study was the addition of validated psychometric instruments alongside symptom assessments. We observed distinct trajectories with all measures showing higher initial prevalence, decreasing over 9 months and with a small proportion with prolonged effects.

PEM was reported by almost half of participants early in their recovering from COVID-19 and fell substantially over the following 3 months. However, it is also clear that there was a smaller cohort in whom PEM continued to occur for several months following initial infection. Most research assessing PEM has focused on individuals with Long-COVID, which explains prevalence exceeding 70% in some cohorts.^44^,^46^ Cognitive function demonstrated a gradual improvement over 9 months, yet a subset of participants had persistent cognitive issues. Few studies have assessed cognitive function in the acute recovery phase, although cognitive dysfunction is a key marker of Long-COVID.^9 22^ A recent meta-analysis did report significant reductions in executive function, attention and working memory.^47^ However, that analysis included only 5 studies and 290 people with Long-COVID. The present data extend this work by demonstrating that cognitive dysfunction assessed using a validated instrument is a common feature of the acute COVID-19 response, which can persist for many months.

EQ5D assessments indicated minimal mobility or self-care limitations post-infection, though about half of participants reported experiencing anxiety, depression and pain. Previous work has detailed more severe or similar long-term health-related quality of life outcomes. However, these reports have not been in cohorts of non-hospitalised participants.^48 49^ Similarly, breathlessness was initially common and fell substantially within 3 months, with a subset experiencing persistent moderate breathlessness. Again, long-term breathlessness has rarely been studied in non-hospitalised patients. Studies in mixed or hospitalised cohorts have reported with higher^50^ or similar proportions but more severe dyspnoea^40^ or dyspnoea. Pain assessments revealed a quarter of participants reported some degree of pain initially with prevalence falling over time. Again, comparison is difficult as previous studies have focused on hospitalised cohorts reporting higher prevalence of pain.^43 51^ The present study also reported pain abated more slowly in older participants, though the effect size was small.

### Strengths and limitations

The primary strength of the present study is the use of validated instruments alongside symptom counts, at a frequency which enabled monthly tracking. This provided a granular view of recovery trajectories from acute COVID-19 infection. Moreover, we purposefully focused on non-hospitalised individuals who have been less well represented in the COVID-19 recovery literature. There are some limitations that should also be considered. Our reliance on mobile technology and social media for recruitment may explain the lack of older participants; thus, findings herein may not apply to those over 70 years of age. This is a distinct limitation given the impact of acute COVID-19 infection on the over 70s in the knowledge that 14% of the Scottish population are over 70 years of age.^52^ Second, our inclusion criteria was within 10 weeks of a self-reported positive test for COVID-19 (the NICE guidelines on what constituted acute, post-acute and persistent symptoms were still being developed when this study commenced), 10 weeks falls within the subacute phase and those people may be more likely to progress into the chronic phase than those who have no symptoms. As such, two potential sampling biases may have occurred. Those that consider themselves having ongoing symptoms participated, resulting in a selection bias, and those with ongoing symptoms but no positive test may have signed up to take part resulting in self-report bias. Our sample demographics speak to this, as 78% of our sample was female but only 51% of the Scottish population is female and Long-COVID is known to disproportionately affect females.^18 53^ Indeed, the use of snowball sampling may have biased the sample further as those with prolonged symptoms highlighted the study specifically to others with similar conditions. Third, excluding individuals with insufficient English proficiency may further limit generalisability, as these individuals may differ in health-seeking behaviours, resource access or cultural attitudes. This exclusion could disproportionately affect non-native speakers, overlapping with underserved groups and potentially perpetuating health disparities. That being said, in the last census, 98.6% of people in Scotland aged 3 and over spoke English.^52^ Fourth, it is difficult to separate those with Long-COVID (caused by an acute COVID-19 infection) from those with post-vaccine syndrome,^54^ or to give its colloquial term, ‘Long Vax(x)’.^55^ Indeed, Arjun *et al*^56^ noted a greater risk (adjusted OR of 2.32) of Long-COVID symptoms in those with two vaccine doses. As 99% of our participants were vaccinated, it is possible that a proportion of people reporting symptoms at 9 months were because of Long Vaxx as opposed to Long-COVID. Penultimately, our list of symptoms was evidence based^9^ and developed with our PPI group (Long-COVID Scotland); presenting participants with a pre-defined list of Long-COVID symptoms may have limited the range or specificity of symptoms reported by participants. Finally, reporting of new infections was low and the possibility of under-reporting cannot be discounted. At that time, 1–2% of the Scottish population was testing positive for COVID-19.^57^ If under-reporting was present, re-infections may have contributed to persistent or re-occurring symptoms.

### Conclusions and future directions

In conclusion, around a third of individuals had no, or limited, symptoms following infection with COVID-19. Of those with symptoms, most recovered over the subsequent months, often much more slowly than from other viral infections. A small proportion (~3%) had ongoing symptoms at the end of the 9-month follow-up. We would resist the temptation to only consider those with ongoing symptoms at the end of follow-up as having ‘true’ Long-COVID however as individuals who recovered slowly still meet the definition of Long-COVID^58^ and experienced debilitating symptoms for several months after infection alongside a prolonged recovery. Future research may wish to identify risk factors that increase Long-COVID propensity, and of course pharmacological^59^,^61^ and non-pharmacological^62^,^64^ interventions to treat Long-COVID.

## supplementary material

10.1136/bmjopen-2024-086646 online supplemental file 1

## Data Availability

Data are available upon reasonable request. All data relevant to the study are included in the article or uploaded as supplementary information.

## References

[1] Wong MK, Brooks DJ, Ikejezie J (2023). COVID-19 Mortality and Progress Toward Vaccinating Older Adults - World Health Organization, Worldwide, 2020-2022. MMWR Morb Mortal Wkly Rep.

[2] Baudin M, Hickey J, Mercier J (2023). COVID-19 vaccine-associated mortality in the Southern Hemisphere.

[3] Sy W (2024). Excess Deaths in the United Kingdom: Midazolam and Euthanasia in the COVID-19 Pandemic.

[4] Tsai P-H, Lai W-Y, Lin Y-Y (2021). Clinical manifestation and disease progression in COVID-19 infection. J Chin Med Assoc.

[5] Carfì A, Bernabei R, Landi F (2020). Persistent Symptoms in Patients After Acute COVID-19. JAMA.

[6] Savarraj JPJ, Burkett AB, Hinds SN (2021). Pain and Other Neurological Symptoms Are Present at 3 Months After Hospitalization in COVID-19 Patients. *Front Pain Res (Lausanne*).

[7] Ward H, Cooke G, Whitaker M (2021). REACT-2 round 5: increasing prevalence of sars-cov-2 antibodies demonstrate impact of the second wave and of vaccine roll-out in england. Infectious Diseases (except HIV/AIDS).

[8] Krumholz HM (2013). Post-Hospital Syndrome – A Condition of Generalized Risk. N Engl J Med.

[9] Hayes LD, Ingram J, Sculthorpe NF (2021). More Than 100 Persistent Symptoms of SARS-CoV-2 (Long COVID): A Scoping Review. Front Med.

[10] Davis HE, Assaf GS, McCorkell L (2021). Characterizing long COVID in an international cohort: 7 months of symptoms and their impact. EClinMed.

[11] Michelen M, Manoharan L, Elkheir N (2021). Characterising long COVID: a living systematic review. BMJ Glob Health.

[12] Li J, Zhou Y, Ma J (2023). The long-term health outcomes, pathophysiological mechanisms and multidisciplinary management of long COVID. *Sig Transduct Target Ther*.

[13] Munblit D, Nicholson T, Akrami A (2022). A core outcome set for post-COVID-19 condition in adults for use in clinical practice and research: an international Delphi consensus study. Lancet Respir Med.

[14] Gross M, Lansang NM, Gopaul U (2023). What Do I Need to Know About Long-Covid-related Fatigue, Brain Fog, and Mental Health Changes?. Arch Phys Med Rehabil.

[15] Aiyegbusi OL, Hughes SE, Turner G (2021). Symptoms, complications and management of long COVID: a review. J R Soc Med.

[16] Ladds E, Rushforth A, Wieringa S (2020). Persistent symptoms after Covid-19: qualitative study of 114 “long Covid” patients and draft quality principles for services. BMC Health Serv Res.

[17] Kingstone T, Taylor AK, O’Donnell CA (2020). Finding the “right” GP: a qualitative study of the experiences of people with long-COVID. BJGP Open.

[18] Bai F, Tomasoni D, Falcinella C (2022). Female gender is associated with long COVID syndrome: a prospective cohort study. Clin Microbiol Infect.

[19] Blomberg B, Mohn KG-I, Brokstad KA (2021). Long COVID in a prospective cohort of home-isolated patients. Nat Med.

[20] Tran V-T, Porcher R, Pane I (2022). Course of post COVID-19 disease symptoms over time in the ComPaRe long COVID prospective e-cohort. Nat Commun.

[21] Thompson EJ, Williams DM, Walker AJ (2022). Long COVID burden and risk factors in 10 UK longitudinal studies and electronic health records. Nat Commun.

[22] O’Mahoney LL, Routen A, Gillies C (2023). The prevalence and long-term health effects of Long Covid among hospitalised and non-hospitalised populations: A systematic review and meta-analysis. E Clin Med.

[23] Munblat D, Nicholson T, Williamson P (2021). Personal Communication: Discussion of the initial outcomes of Long-COVID Core Outcome Set (COS).

[24] Tong A, Baumgart A, Evangelidis N (2021). Core Outcome Measures for Trials in People With Coronavirus Disease 2019: Respiratory Failure, Multiorgan Failure, Shortness of Breath, and Recovery. Crit Care Med.

[25] Huo T, Guo Y, Shenkman E (2018). Assessing the reliability of the short form 12 (SF-12) health survey in adults with mental health conditions: a report from the wellness incentive and navigation (WIN) study. Health Qual Life Outcomes.

[26] Cotler J, Holtzman C, Dudun C (2018). A Brief Questionnaire to Assess Post-Exertional Malaise. *Diagnostics (Basel*).

[27] Shipston-Sharman O, Hoeritzauer I, Edwards M (2019). Screening for functional neurological disorders by questionnaire. J Psychosom Res.

[28] Yorke J, Russell A-M, Swigris J (2011). Assessment of dyspnea in asthma: validation of The Dyspnea-12. J Asthma.

[29] Vartiainen P, Mäntyselkä P, Heiskanen T (2017). Validation of EQ-5D and 15D in the assessment of health-related quality of life in chronic pain. Pain.

[30] Löwe B, Wahl I, Rose M (2010). A 4-item measure of depression and anxiety: validation and standardization of the Patient Health Questionnaire-4 (PHQ-4) in the general population. J Affect Disord.

[31] Gruber-Baldini AL, Velozo C, Romero S (2017). Validation of the PROMIS^®^ measures of self-efficacy for managing chronic conditions. Qual Life Res.

[32] van Oirschot P, Heerings M, Wendrich K (2020). Symbol Digit Modalities Test Variant in a Smartphone App for Persons With Multiple Sclerosis: Validation Study. JMIR Mhealth Uhealth.

[33] Gabrio A, Plumpton C, Banerjee S (2022). Linear mixed models to handle missing at random data in trial-based economic evaluations. Health Econ.

[34] NICE (2020). Overview | COVID-19 rapid guideline: managing the long-term effects of COVID-19 | Guidance.

[35] Munblit D, Simpson F, Mabbitt J (2022). Legacy of COVID-19 infection in children: long-COVID will have a lifelong health/economic impact. Arch Dis Child.

[36] Carroll N, Sadowski A, Laila A (2020). The Impact of COVID-19 on Health Behavior, Stress, Financial and Food Security among Middle to High Income Canadian Families with Young Children. Nutrients.

[37] Office for National Statistics People not in work.

[38] Naik S, Haldar SN, Soneja M (2021). Post COVID-19 sequelae: A prospective observational study from Northern India. DD&T.

[39] Mohiuddin Chowdhury ATM, Karim MR, Ali MA (2021). Clinical Characteristics and the Long-Term Post-recovery Manifestations of the COVID-19 Patients-A Prospective Multicenter Cross-Sectional Study. Front Med (Lausanne).

[40] Evans RA, Leavy OC, Richardson M (2022). Clinical characteristics with inflammation profiling of long COVID and association with 1-year recovery following hospitalisation in the UK: a prospective observational study. Lancet Respir Med.

[41] Sigfrid L, Drake TM, Pauley E (2021). Long Covid in adults discharged from UK hospitals after Covid-19: A prospective, multicentre cohort study using the ISARIC WHO Clinical Characterisation Protocol. *Lancet Reg Health Eur*.

[42] Office for National Statistics (2022). Prevalence of ongoing symptoms following coronavirus (COVID-19) infection in the UK.

[43] Taş A, Baloğlu M (2023). Post-COVID syndrome and pain perception in outpatients with COVID-19. Eur Rev Med Pharmacol Sci.

[44] Jason LA, Dorri JA (2022). ME/CFS and Post-Exertional Malaise among Patients with Long COVID. Neurol Int.

[45] Mclaughlin M, Cerexhe L, Macdonald E (2023). A Cross-Sectional Study of Symptom Prevalence, Frequency, Severity, and Impact of Long-COVID in Scotland: Part I. Am J Med.

[46] Twomey R, DeMars J, Franklin K (2022). Chronic Fatigue and Postexertional Malaise in People Living With Long COVID: An Observational Study. Phys Ther.

[47] Crivelli L, Palmer K, Calandri I (2022). Changes in cognitive functioning after COVID-19: A systematic review and meta-analysis. Alzheimers Dement.

[48] Kim Y, Kim S-W, Chang H-H (2022). One Year Follow-Up of COVID-19 Related Symptoms and Patient Quality of Life: A Prospective Cohort Study. Yonsei Med J.

[49] Betschart M, Rezek S, Unger I (2021). One year follow-up of physical performance and quality of life in patients surviving COVID-19: a prospective cohort study. Swiss Med Wkly.

[50] Evans RA, McAuley H, PHOSP-COVID Collaborative Group (2021). Physical, cognitive and mental health impacts of COVID-19 following hospitalisation – a multi-centre prospective cohort study. Infect Dis (except HIV/AIDS).

[51] Karaarslan F, Güneri FD, Kardeş S (2022). Long COVID: rheumatologic/musculoskeletal symptoms in hospitalized COVID-19 survivors at 3 and 6 months. Clin Rheumatol.

[52] Scotland’s Census Scotland’s Census at a glance: Languages.

[53] Ortona E, Malorni W (2022). Long COVID: to investigate immunological mechanisms and sex/gender related aspects as fundamental steps for tailored therapy. Eur Respir J.

[54] Ablin JN, Shoenfeld Y, Buskila D (2006). Fibromyalgia, infection and vaccination: two more parts in the etiological puzzle. J Autoimmun.

[55] Tindle R (2024). Long COVID: Sufferers can take heart. *Aust J Gen Pract*.

[56] Arjun MC, Singh AK, Pal D (2022). Characteristics and predictors of Long COVID among diagnosed cases of COVID-19. PLoS One.

[57] Public Health Scotland (2022). COVID-19 statistical report.

[58] Venkatesan P (2021). NICE guideline on long COVID. Lancet Respir Med.

[59] Wu X, Xiang M, Jing H (2024). Damage to endothelial barriers and its contribution to long COVID. Angiogenesis.

[60] Jarrott B, Head R, Pringle KG (2022). “LONG COVID”-A hypothesis for understanding the biological basis and pharmacological treatment strategy. Pharmacol Res Perspect.

[61] Trinh NT, Jödicke AM, Català M (2024). Effectiveness of COVID-19 vaccines to prevent long COVID: data from Norway. Lancet Respir Med.

[62] Sanal-Hayes NEM, Mclaughlin M, Mair JL (2024). ‘Pacing’ for management of myalgic encephalomyelitis/chronic fatigue syndrome (ME/CFS): a systematic review and meta-analysis. Fatigue.

[63] Sanal-Hayes NEM, Mclaughlin M, Hayes LD (2023). A scoping review of “Pacing” for management of Myalgic Encephalomyelitis/Chronic Fatigue Syndrome (ME/CFS): lessons learned for the long COVID pandemic. J Transl Med.

[64] Vernon SD, Hartle M, Sullivan K (2023). Post-exertional malaise among people with long COVID compared to myalgic encephalomyelitis/chronic fatigue syndrome (ME/CFS). Work.

