# Supplementary material for: Tracking Persistent Symptoms in Scotland (TraPSS): a longitudinal prospective cohort study of COVID-19 recovery after mild acute infection
Source: BMJ Open. 2025 Jan 15;15(1):e086646. doi: 10.1136/bmjopen-2024-086646 (PMC11751823; doi:10.1136/bmjopen-2024-086646)
Supplement: online supplemental file 1 [file bmjopen-15-1-s001.pdf]

Supplementary table 1. Participant Demographics

	**Combined (n = 287)**	**Males (n = 63)**	**Females (n = 224)**
Age (years; Mean ± SD; range)	43.4 ± 12.4 (20-72)	43.5 ± 12.4 (20-72)	43.4 ± 11.9 (21-69)
Underlying health conditions (%)			
Yes	116		
Asthma	41	7	34
Breathing problems or other lung conditions	1	1	0
Cancer	6	3	3
Diabetes	10	4	6
Heart disease	5	3	2
Hypertension	17	9	8
Kidney disease	1	0	1
Other	60	12	48
Vaccination Status (*n*)			
Yes - at least 2 doses plus booster	234	52	182
Yes - at least 3 doses	61	9	52
Yes - at least 2 doses	276	54	218
Yes - at least 1 dose	284	62	222
No	3	1	2
