# Supplementary material for: Tracking Persistent Symptoms in Scotland (TraPSS): a longitudinal prospective cohort study of COVID-19 recovery after mild acute infection
Source: BMJ Open. 2025 Jan 15;15(1):e086646. doi: 10.1136/bmjopen-2024-086646 (PMC11751823; doi:10.1136/bmjopen-2024-086646)
Supplement: Supplementary file 2 [file bmjopen-2024-086646.reviewer_comments.pdf]

PEER REVIEW HISTORY

BMJ Open publishes all reviews undertaken for accepted manuscripts. Reviewers are asked to complete a checklist review form and are provided with free text boxes to elaborate on their assessment. These free text comments are reproduced below.

ARTICLE DETAILS

Title (Provisional)

Tracking Persistent Symptoms in Scotland (TraPSS): A Longitudinal Prospective Cohort Study of COVID-19 Recovery After Mild Acute Infection

Authors

Sculthorpe, Nicholas; McLaughlin, Marie; Cerexhe, Luke; Macdonald, Eilidh; Dello Iacono, Antonio; Sanal-Hayes, Nilihan; Ingram, Joanne; Meach, Rachel; Carless, David; Ormerod, Jane; Hayes, Lawrence D

VERSION 1 - REVIEW

**Reviewer**	**1**
**Name**	**Degens, Hans**
**Affiliation**	**Manchester Metropolitan University, School of Healthcare Science**
**Date**	**19-May-2024**
**COI**	**None**

In this study it was found, using an Ap, that long-COVID was more prevalent in older age and women.

The paper is well-written and has a nice flow.

I suggest the authors use the term ‘sex’ throughout the manuscript rather than ‘gender’ as ‘gender’ is a social construct.

It is interesting to note that post-(COVID-19) vaccination syndrome has similar effects as described for long COVID (Krumholz et al., medRxiv, 2023: 10.1101/2023.11.09.23298266) and even before COVID there was evidence that vaccinations can cause long-COVID-like symptoms (Ablin et al., J Autoimmun, 2006). In addition, Arjun et al., (PlosOne, 2022) write:

‘Statistically significant predictors of Long COVID at four weeks of follow-up were— Pre-existing medical conditions (Adjusted Odds ratio (aOR) = 2.00, 95% CI: 1.16,3.44), having a higher number of symptoms during acute phase of COVID-19 disease (aOR = 11.24, 95% CI: 4.00,31.51), two doses of COVID-19 vaccination (aOR = 2.32, 95% CI: 1.17,4.58),…,’.

In another paper it is written: ‘At present, public health officials are flying blind when it comes to long COVID and vaccination’ and ‘There is concern that COVID-19 vaccination per se might contribute to long COVID, giving rise to the colloquial term ‘Long Vax(x)’ (Tindle, AJGP 53(4):238-240, 2024)

In another paper it is written: Further reports indicating vaccines may cause long-COVID symptoms are …. (Couzin-Frankel & Vogel, Science 375 issue 6579:364-366, 2022)

The question therefore is to what extent the observed phenomena (long-COVID) is therefore an effect of the COVID infection or a consequence of vaccination. The design of the study is inadequate to disentangle these factors as virtually all participants, bar 3, were vaccinated.

Introduction

Page 4 opening sentence: How do we know that >6.9 million people died worldwide of COVID infection, rather than of other causes? Note that in the UK, for instance, any person dying within 28 days of a COVID+ test was considered a COVID death, where the cause most likely was otherwise. A Freedom of Information request to the Office of National Statistics showed (that at the time of the request; Jan 2022) just 17,000 people had only COVID on their death certificate of the allegedly 127,000 COVID-related deaths. Note that in the UK around 10,000 people a week die. If we then know that 74% of the population have corrective eye wear or had laser surgery (https://wcb-ccd.org.uk/library/beh_report_final_(1).pdf), we can assume that around 7400 people are registered dying while wearing glasses, but they did not die of the glasses. It is also unlikely that the 10,000 people died because they ate a potato or potato-related product in the 28 days before death. It is important that dying with a COVID+ test within 28 days before death does not mean they died of COVID. Likewise, a COVID+ test and subsequent long-COVID symptoms are not necessarily related, particularly if we know that vaccines can cause the same problems. Another paper indeed reported:

‘This paper shows that the UK spike in deaths, wrongly attributed to COVID-19 in April 2020, was not due to SARS-CoV-2 virus, which was largely absent, but was due to the widespread use of Midazolam injections which were statistically very highly correlated (coefficient over 90 percent) with excess deaths in all regions of England during 2020. Importantly, excess deaths remained elevated following mass vaccination in 2021, but were statistically uncorrelated to COVID vaccination, while remaining significantly correlated to Midazolam injections’ (Sy, Med & Clin Res 9(2):1-21, 2024; DOI: 10.13140/RG.2.2.13654.42560).

And another report again tells us:

‘We quantify the overall all-ages vaccine-dose fatality rate (vDFR) for the 17 countries to be (0.126 ± 0.004) %, which would imply 17.0 ± 0.5 million COVID-19 vaccine deaths worldwide, from 13.50 billion injections up to 2 September 2023. This would correspond to a mass iatrogenic event that killed (0.213 ± 0.006) % of the world population (1 death per 470 living persons, in less than 3 years), and did not measurably prevent any deaths.’ (Rancourt et al.,

2023; COVID-19 vaccine-associated mortality in the Southern Hemisphere – CORRELATION (correlation-canada.org)).

This casts doubt on the statement that the deaths are COVID related, and could well be intervention (Midazolam, a respiratory depressant, in the first paper) or COVID-19 vaccination (the second report) and the same may well apply to long-COVID symptoms.

Last sentence of first paragraph: long-COVID like symptoms were long before COVID recognised as complications post-hospitalisation.

Methods

Major comment: Given the considerations above, it should be recognised that the symptoms could with as much reason be ascribed to COVID vaccination rather than a COVID+ test (note there are also false positives!) can also be an effect of vaccination. You have only 3 unvaccinated. Consider that not all COVID+ develop so-called long-COVID and likewise (same reasoning) not all vaccinated develop 'Long vaccine' or here 'Long-COVID. In other words, it should be recognised that your study can't conclude whether the symptoms derive from the vaccination or infection. If you wanted to be provide more solid evidence for the consequence of infection you should have had equal groups of vaccinated and unvaccinated (that is no vaccine at all). Perhaps you could subdivide the vaccinated in those with 1 vs more than 1 vaccine (I am not sure what the difference is between '3 doses' and '2 doses + booster'; is the booster not the third vaccination (although there could be even more than 3!). This subdivision may give some hint on what the effect of vaccination is.

Another consideration is that the symptoms are self-reported, and perhaps particularly those that consider themselves having symptoms of long-COVID participate, as they are keener than others to know how it develops. This may well be possible, as inclusion within 10 weeks of testing positive for COVID is a long time. This falls within the subacute phase, where those in the subacute phase are more likely to progress into the chronic phase than those who have no symptoms. There may thus be a selection bias in the population studied.

Results

Can you give an indication whether the reduction of work hours is related in one way or another to infection (or vaccination?) symptoms. In other words, are those with most reduced working hours those that have the symptoms the longest? While I appreciate the numbers may be too low, there is perhaps a trend noticeable, or at least plot it, so people can see it.

Is there a correlation between the presence of say loss of appetite and muscle pain, or loss of appetite and headache, or muscle pain and headache, or with loss of smell or taste. Can you perhaps make a graph indicating people with 6, 5, 4, 3, 2, 1 symptom at start and how that relates to longevity of the symptoms and loss of working hours? I also find it a bid strange that the symptoms are 1-7 times a week. This suggests that in some people at day 1 they may have a loss of smell, day 2 they are fine, but day 3 they suffer again. This is rather

peculiar. How can this be; when I have the flu it is not one day off, then on again… Why is fatigue, the most prevalent symptom not panel A? In the text, do refer to the figures and panels.

To what extent is the improvement over time in cognitive function related to a learning effect?

**Reviewer**	**2**
**Name**	**Ferraro, Francesco**
**Affiliation**	**University of Derby, Clinical Exercise and Rehabilitation Research Centre**
**Date**	**28-May-2024**
**COI**	**None**

Thank you for submitting your work.

I found your manuscript very interesting, please find below my comments and considerations.

Introduction.

Page 5, line 5 – remove "novel"

Page 5, line 8 – add space in "damage[2]."

Page 5, line 14 – add a number of participants based on recent reports and data: "13% in select community-based cohorts with laboratory-confirmed COVID-19, to upwards of 71% in hospitalized patients". These data are based only in the UK so we would need to know how many are 13% and 71%?

Page 5, line 31 – the authors made an excellent point in "They also reported nearly 70% of patients received a diagnosis of Long-COVID. However, their high prevalence rate was likely influenced by their data coming from a specific post-COVID outpatient service, with most patients having been both hospitalized and intubated during their acute infection. Consequently, it is unclear how well this kind of prospective data reflects the natural progression of the condition in many people who remained community-dwelling and were never hospitalized during the acute phase." This needs a bit more explanation/consideration. The discussion around Long COVID, with some authors saying that it does not exist, requires attention. I also recommend the authors provide a definition of Long COVID.

Page 5, line 50 – The authors` aims and objectives are clear. But what is the impact of this research? Why is it important?

Methods

Page 6, line 30 – please provide details on how "cognitive function" was assessed

Page 6, line 37 – you mentioned that patients need to have a positive COVID test (page 6, line 29), and then you reported that recruiting was completed online. Please clarify if a positive covid test was then self-claimed by the patients or not.

Discussion

Page 16, line 10 – the authors discussed long covid in the introduction, then reported fatigue elements in their results and discussion, but failed to link these with their introduction. I recommend talking more about long-term COVID and what others have reported in the literature on this matter. The results on fatigue are extremely valuable and have been well discussed by other authors. My recondition would be to contextualise your results with the current literature knowledge.

Limitation

Page 17 – I would suggest that the authors consider their sample of 287 patients. Was this number expected? Where do the authors consider to have a bigger sample size? Given the number of positive COVID-19 patients, it seems quite a small number. Can the authors include their perspectives on these elements in their limitations?

Conclusion

Page 17, line 47 – add space “Long-COVID[37]”

Page 17 – the “future directions” are missing from “Conclusions and future directions”

Page 17, line 40 – it is not clear the impact of this research and the take-home point. The authors should use their conclusion to i) state that they met their aims and objectives, ii) clarify the take-home point (not only for academics) but also for health staff, and iii) demonstrate that their research is making an impact.

References

I feel the above comment would also help to improve the number of references to your work.

**Reviewer**	**3**
**Name**	**Perquin, Magali**
**Affiliation**	**Luxembourg Institute of Health**
**Date**	**03-Jun-2024**
**COI**	**I declare no conflict of interest with this work.**

Please find my comments, questions, and suggestions below, which should be addressed to improve the work. They are listed in the order of appearance in the manuscript (page numbers refer to the BMJ Open PDF):

Line 14 p 5 (Introduction): Please add reference(s) on prevalence ranges (13 to 71%) at the end of the paragraph.

Lines 5 p 6 (Methods) and Line 23 p 21 (Ethics statement): Is it normal to have a discrepancy in the approval number mentioned: 14019 vs 14988?

Line 19 p6 (Patients involvement):

- The acronym PPI should be defined here, rather than later;
- This Patient Involvement paragraph should be written in a more neutral tone. Explicit mentions of co-authors and their roles should be omitted from this section and instead included in the contributors section.

Line 28 p6 (Inclusion criteria): Could you please be more specific about the type of positive COVID-19 test required? Are any techniques acceptable?

Line 48 p6 (Protocol for downloading the app): It is unnecessary to repeat “Once downloaded.”

Line 21-22 p7 (Figure 1. caption): The acronyms Eq5D and VAS should be defined again (as TraPSS

is).

Line 51 p7 (App design): MRC should be defined (Medical Research Council).

Line 9-12 p8 (App design): The description of SDMT could be slightly refined for clarity and conciseness.

Line 27 p8 (Statistical analysis): The presentation of your model is not so clear to me:

Either you mention a typical linear mixed-effects model such as $\text{Outcome}_{in} = \beta 0+\beta 1\cdot \text{time}_{in} + \beta 2\cdot \text{gender}_{i} + \beta 3\cdot \text{age}_{i} + \beta 4\cdot (\text{time}_{in}\cdot \text{gender}_{i}) + \beta 5\cdot (\text{time}_{in}\cdot \text{age}_{i}) + \beta 6\cdot (\text{gender}_{i}\cdot \text{age}_{i}) + \beta 7\cdot (\text{time}_{in}\cdot \text{gender}_{i}\cdot \text{age}_{i}) + (1|\text{Subject}_{i}) + \epsilon_{in}$, to examine the effects of time, age, and gender on the construct of recovery. Or, you use the given model ($\text{Outcome}_{in}=b_{0in}+b_{1-9}\text{time}*b_{4-5}*\text{gender}*\text{age}+\epsilon i$) which is not a traditional linear mixed-effects model. This model presents characteristics more typical of a regression model with complex interactions between time, gender, and age. Can you please clarify this part?

Line 53 p8 (Results): Before the start of results *per se*, a basic scheme summarizing the different steps of recruitment process with numbers of participants, loss to follow-up if any etc. would be appreciated. Moreover, it fits with the STROBE Statement request.

Line 53 p8 (Results): Due to the non-probabilistic nature of the sampling method (snowball sampling), calculating the sample size for the study is indeed challenging. However, could you please explain why 287 participants? Are there some approaches and considerations you use to estimate a sample size needed to achieve certain study objectives?

Line 6 p9 (Infection recovery and change to work): Figure 1 mentioned here refers rather to Figure 2. Please correct this.

Line 7-8 p9 (Infection recovery and change to work): Please correct the sentence "The 3.1% not reporting ‘completely recovered at month 9, reports are of being ‘Mostly recovered’ (2.4%) or ‘about half recovered’ (0.7%)." Do you mean: "The 3.1% not reporting ‘completely recovered’ at month 9, reported being ‘Mostly recovered’ (2.4%) or ‘about half recovered’ (0.7%)."?

Line 34 p9 (Figure 2.): It seems that there is an inconsistency regarding the number of months indicated: it is stated as “9 months” in the text, figure title, and subsequent graphs, whereas in Figure 2, it is shown as 10 months.

Line 12 p10 (Symptom frequency): The sentence “64.2% of participants reported some level of fatigue at baseline.” is already written just above (1st line, 1st paragraph).

Line 15 p10 (Symptom frequency): An inconsistency seems to exist here: in "(decreasing from 37.9% at baseline)". Isn't it 36.9% at baseline (and not 37.9%) as mentioned in the previous paragraph?

Line 28 p10 (Symptom frequency): Is the sign of the estimate (0.09) correct here?

P11-12 (Figure 3): It would be preferable to have all the graphs on the same page/figure. If this is not possible, please provide a title, caption, and figure number for each page (dissociating A to F from G to N).
Please correct the typo “msucle pain (B)”.

Line 15-16 p13 (EQ5D): Please use “Impairments in mobility” and “Impairments in self-care” instead of “mobility” and “self-care” to improve clarity. Same suggestion in Figure 4 sub-titles.

P13 (Figure 4): Please provide a caption for Figure 4 that explains the meaning of 0, 1, 2, 3, 4, etc. Also, check Graph 4C for 'legend'.

Line 7 p14 (Dyspnea, VAS pain, anxiety/depression, and self-efficacy): Please clarify the difference between anxiety/depression as presented here and anxiety and depression measured with the EQ5D, as shown in the previous paragraph.

Line 36-37 p14 (Cognitive function): Please revise this sentence “This was similar for total time to correct answers at 29.7% at baseline to 90.4% at month 9.”

P15 (Figure 5): Please revise the sub-titles G, H, I and refer to SDMT (for example “SDMT: Number of incorrect answers”).

P17 (Strengths and limitations):
Line 27: Please correct the typo “focussed”
Line 30: Please correct “yrs”
Line 29-30: Additional potential bias (other than demographic ones) should be mentioned and discussed, such as self-selection bias, network bias etc., all of which can affect the generalizability of the study findings.

VERSION 1 - AUTHOR RESPONSE

Reviewer: 1

Prof. Hans Degens, Manchester Metropolitan University

Comments to the Author:

In this study it was found, using an Ap, that long-COVID was more prevalent in older age and women.

The paper is well-written and has a nice flow.

Thank you for this positive comment.

I suggest the authors use the term 'sex' throughout the manuscript rather than 'gender' as 'gender' is a social construct.

Thank you for this comment and we agree gender is a social construct. However, using the SAGER guidelines (https://researchintegrityjournal.biomedcentral.com/articles/10.1186/s41073-016-0007-6), which entail that terminology be used as they were presented to the participants in the study, we asked participants their gender. Thus, they reported how they identified rather than their sex at birth.

It is interesting to note that post-(COVID-19) vaccination syndrome has similar effects as described for long COVID (Krumholz et al., medRxiv, 2023: 10.1101/2023.11.09.23298266) and even before COVID there was evidence that vaccinations can cause long-COVID-like symptoms (Ablin et al., J Autoimmun, 2006). In addition, Arjun et al., (PlosOne, 2022) write:

'Statistically significant predictors of Long COVID at four weeks of follow-up were— Pre-existing medical conditions (Adjusted Odds ratio (aOR) = 2.00, 95% CI: 1.16,3.44), having a higher number of symptoms during acute phase of COVID-19 disease (aOR = 11.24, 95% CI: 4.00,31.51), two doses of COVID-19 vaccination (aOR = 2.32, 95% CI: 1.17,4.58),...,'.

In another paper it is written: 'At present, public health officials are flying blind when it comes to long COVID and vaccination' and 'There is concern that COVID-19 vaccination per se might contribute to long COVID, giving rise to the colloquial term 'Long Vax(x)' (Tindle, AJGP 53(4):238-240, 2024)

In another paper it is written: Further reports indicating vaccines may cause long-COVID symptoms are .... (Couzin-Frankel & Vogel, Science 375 issue 6579:364-366, 2022)

The question therefore is to what extent the observed phenomena (long-COVID) is therefore an effect of the COVID infection or a consequence of vaccination. The design of the study is inadequate to disentangle these factors as virtually all participants, bar 3, were vaccinated.

We thank R1 for this comment. Indeed, it is possible that participants reporting long COVID symptoms were in fact post-vaccine symptoms. We asked participants to report vaccine status on enrolment, and also additional vaccines as and when they occurred. However, due to heterogeneity of vaccine status (and even reporting), we are unable to explore the effect of vaccines on temporal symptom changes. We have added this as a limitation as follows:

'Secondly, it is difficult to separate those with Long-COVID (caused by an acute COVID-19 infection) from those with post-vaccine syndrome [36], or to give its colloquial term, 'Long Vax(x)' [37]. Indeed, Arjun et al. [38] noted a greater risk (adjusted odds ratio of 2.32) of Long-COVID symptoms in those with two vaccine doses. As 99% of our participants were vaccinated, it is possible that a proportion of people reporting symptoms at 9 months were because of Long Vaxx as opposed to Long-COVID.'

Introduction

Page 4 opening sentence: How do we know that >6.9 million people died worldwide of COVID infection, rather than of other causes? Note that in the UK, for instance, any person dying within 28 days of a COVID+ test was considered a COVID death, where the cause most likely was otherwise. A Freedom of Information request to the Office of National Statistics showed (that at the time of the request; Jan 2022) just 17,000 people had only COVID on their death certificate of the allegedly 127,000 COVID-related deaths. Note that in the UK around 10,000 people a week die. If we then know that 74% of the population have corrective eye wear or had laser surgery (https://wcb-ccd.org.uk/library/beh_report_final_(1).pdf), we can assume that around 7400 people are registered dying while wearing glasses, but they did not die of the glasses. It is also unlikely that the 10,000 people died because they ate a potato or potato-related product in the 28 days before death. It is important that dying with a COVID+ test within 28 days before death does not mean they died of COVID. Likewise, a COVID+ test and subsequent long-COVID symptoms are not necessarily related, particularly if we know that vaccines can cause the same problems. Another paper indeed reported:

‘This paper shows that the UK spike in deaths, wrongly attributed to COVID-19 in April 2020, was not due to SARS-CoV-2 virus, which was largely absent, but was due to the widespread use of Midazolam injections which were statistically very highly correlated (coefficient over 90 percent) with excess deaths in all regions of England during 2020. Importantly, excess deaths remained elevated following mass vaccination in 2021, but were statistically uncorrelated to COVID vaccination, while remaining significantly correlated to Midazolam injections’ (Sy, Med & Clin Res 9(2):1-21, 2024; DOI: 10.13140/RG.2.2.13654.42560).

And another report again tells us:

‘We quantify the overall all-ages vaccine-dose fatality rate (vDFR) for the 17 countries to be (0.126 ± 0.004) %, which would imply 17.0 ± 0.5 million COVID-19 vaccine deaths worldwide, from 13.50 billion injections up to 2 September 2023. This would correspond to a mass iatrogenic event that killed (0.213 ± 0.006) % of the world population (1 death per 470 living persons, in less than 3 years), and did not measurably prevent any deaths.’ (Rancourt et al., 2023; COVID-19 vaccine-associated mortality in the Southern Hemisphere – CORRELATION (correlation-canada.org)).

This casts doubt on the statement that the deaths are COVID related, and could well be intervention (Midazolam, a respiratory depressant, in the first paper) or COVID-19 vaccination (the second report) and the same may well apply to long-COVID symptoms.

Thank you for these valuable resources. We have tempered our initial sentence as follows: ‘Since its emergence in late 2019, the novel coronavirus, severe acute respiratory syndrome coronavirus 2 (SARS-CoV-2), has rapidly spread across the globe, with >760 million confirmed infections [1]. Although there is considerable disagreement in the exact number of deaths from COVID-19 [2,3], some reports have estimated the number as >6.9 million worldwide [1].’

Last sentence of first paragraph: long-COVID like symptoms were long before COVID recognised as complications post-hospitalisation.

We agree with R1 about this. LC-like symptoms were around long before COVID-19 in the post-acute phase. We have added the following sentence:

"It is worth noting however, that even before COVID-19, nearly one fifth of patients discharged from a hospital develop an acute medical problem within the subsequent 30 days that cause another hospitalization [8]."

Methods

Major comment: Given the considerations above, it should be recognised that the symptoms could with as much reason be ascribed to COVID vaccination rather than a COVID+ test (note there are also false positives!) can also be an effect of vaccination. You have only 3 unvaccinated. Consider that not all COVID+ develop so-called long-COVID and likewise (same reasoning) not all vaccinated develop 'Long vaccine' or here 'Long-COVID. In other words, it should be recognised that your study can't conclude whether the symptoms derive from the vaccination or infection. If you wanted to be provide more solid evidence for the consequence of infection you should have had equal groups of vaccinated and unvaccinated (that is no vaccine at all). Perhaps you could subdivide the vaccinated in those with 1 vs more than 1 vaccine (I am not sure what the difference is between '3 doses' and '2 doses + booster'; is the booster not the third vaccination (although there could be even more than 3!). This subdivision may give some hint on what the effect of vaccination is.

We thank R1 for their comment and agree we cannot disentangle long-COVID from long-vaxx. Participants in the present study also changed vaccine status throughout the study (i.e. receiving an additional dose or a 'booster'), so splitting participant into discrete groups is impossible.

We have added the following limitation to acknowledge this:

'Secondly, it is difficult to separate those with Long-COVID (caused by an acute COVID-19 infection) from those with post-vaccine syndrome [36], or to give its colloquial term, 'Long Vax(x)' [37]. Indeed, Arjun et al. [38] noted a greater risk (adjusted odds ratio of 2.32) of Long-COVID symptoms in those with two vaccine doses. As 99% of our participants were vaccinated, it is possible that a proportion of people reporting symptoms at 9 months were because of Long Vaxx as opposed to Long-COVID.'

Regarding the difference between '3 doses' and '2 doses + booster', for most people, the initial vaccination includes two shots (around a month apart) to achieve initial immunity. After that, they received a booster to re-establish immunity, which may have waned over time (timing of booster varied). Those considered immuno-compromised or clinically extremely vulnerable received 3 (with dose three, usually 8 weeks after dose 2) doses as part of the initial immunity protection. These groups may also have had a booster. We differentiated on the app onboarding for clarity (e.g. it would have been confusing for someone who had had 3 doses if we only gave options for 1, 2, and booster).

Another consideration is that the symptoms are self-reported, and perhaps particularly those that consider themselves having symptoms of long-COVID participate, as they are keener than others to know how it develops. This may well be possible, as inclusion within 10 weeks of testing positive for COVID is a long time. This falls within the subacute phase, where those in the subacute phase are more likely to progress into the chronic phase than those who have no symptoms. There may thus be a selection bias in the population studied.

We thank R1 for this suggestion. At the time, NICE criteria for the phases were still in development so we opted to be cautious with our inclusion criteria. However, to reflect this limitation, we have updated the discussion as follows: *'Secondly, following on from the previous limitation around selection bias, as our inclusion criteria was within 10 weeks of testing positive for COVID-19 (the NICE guidelines on what constituted acute, post-acute, persistent symptoms were still being developed when this study commenced), 10 weeks falls within the subacute phase, and those people may be more likely to progress into the chronic phase than those who have no symptoms. As such, perhaps those that consider themselves having ongoing symptoms participated, resulting in a selection bias.'*

Results

Can you give an indication whether the reduction of work hours is related in one way or another to infection (or vaccination?) symptoms. In other words, are those with most reduced working hours those that have the symptoms the longest? While I appreciate the numbers may be too low, there is perhaps a trend noticeable, or at least plot it, so people can see it.

We thank R1 for their suggestion and we understand the point that if would be interesting to know whether those with the most symptoms have the most reduced work hours. We agree, this would seem logical. However, this was not the *a priori* aim of the present study to determine this. Specifically, our aims were *'(1) to evaluate the natural history of symptoms post-infection, (2) to detect the proportion of people who have persistent symptoms, (3) to identify the most common symptoms associated with COVID-19 recovery and their relative frequencies, and (4) to identify associations of gender and age with symptom recovery.'* We have previously reported correlations in our previous cross-sectional work (https://www.amjmed.com/article/S0002-9343(23)00460-6/fulltext and https://www.amjmed.com/article/S0002-9343(23)00461-8/fulltext), observing more severe symptoms were associated with more severe impact on work and study. Secondly, and an important point to consider is that we have 10 time points (month 0-9) and tens of dependent variables. Each of these is scored on a 5-point to 8-point Likert scale (dependent on the validated instrument). As such, running these analyses would result in hundreds of correlation coefficients. We do not think this is good scientific practice. Similarly, in terms of plotting the relationship(s), we are at the limit of figure uploads, so adding x many new figures is not possible.

We are happy to defer back to R1 for a discussion around this point, or to the editor for input.

Is there a correlation between the presence of say loss of appetite and muscle pain, or loss of appetite and headache, or muscle pain and headache, or with loss of smell or taste. Can you perhaps make a graph indicating people with 6, 5, 4, 3, 2, 1 symptom at start and how that relates to longevity of the symptoms and loss of working hours?

I also find it a bid strange that the symptoms are 1-7 times a week. This suggests that in some people at day 1 they may have a loss of smell, day 2 they are fine, but day 3 they suffer again. This is rather peculiar. How can this be; when I have the flu it is not one day off, then on again... Why is fatigue, the most prevalent symptom not panel A? In the text, do refer to the figures and panels.

We thank R1 for these comments and will address them separately:

"Is there a correlation between the presence of say loss of appetite and muscle pain, or loss of appetite and headache, or muscle pain and headache, or with loss of smell or taste. Can you perhaps make a graph indicating people with 6, 5, 4, 3, 2, 1 symptom at start and how that relates to longevity of the symptoms and loss of working hours?"

We thank R1 for their suggestion and we will respond in the same manner as to the above recommendation. We have previously reported correlations in our previous cross-sectional work (https://www.amjmed.com/article/S0002-9343(23)00460-6/fulltext and https://www.amjmed.com/article/S0002-9343(23)00461-8/fulltext), observing more severe symptoms were associated with more severe impact on work and study. Secondly, and an important point to consider is that we have 10 time points (month 0-9) and tens of dependent variables. Each of these is scored on a 5-point to 8-point Likert scale (dependent on the validated instrument). As such, running these analyses would result in hundreds of correlation coefficients. We do not think this is good scientific practice. Similarly, in terms of plotting the relationship(s), we are at the limit of figure uploads, so adding x many new figures is not possible.

Like above, we are happy to defer back to R1 for a discussion around this point, or to the editor for input.

"I also find it a bid strange that the symptoms are 1-7 times a week. This suggests that in some people at day 1 they may have a loss of smell, day 2 they are fine, but day 3 they suffer again. This is rather peculiar. How can this be; when I have the flu it is not one day off, then on again... Why is fatigue, the most prevalent symptom not panel A? In the text, do refer to the figures and panels."

The nature of long COVID is that one day they can feel OK one day and the next day have a symptom. We know that algias and fatigue particularly can come and go.

We have now referred to figures and panels within text. Apologies of this vagary.

To what extent is the improvement over time in cognitive function related to a learning effect?

We agree with R1 it is possible that cognitive function improvement may have been partially a learning effect. However, if learning effect were the sole contributor, one would expect a plateau after some months (2-3?). Indeed, Tung et al

(https://doi.org/10.3109/09638288.2015.1111438) reported the tablet-SDMT (T-SDMT) exhibited a trivial (d=0.12) but significant (p≦0.015) practice effect. However, this was two weeks apart and only conducted twice. The improvement in the T-SDMT was 4.7%and the minimal detectable change was 6.7%. We therefore suggest changes reported in our manuscript were likely due to something other than learning effect, or more precisely exceeded the differences exceeded changes due solely to learning effect.

Reviewer: 2

Dr. Francesco Ferraro, University of Derby

Comments to the Author:

Thank you for submitting your work.

I found your manuscript very interesting, please find below my comments and considerations.

Thank you for your positive comment.

Introduction.

Page 5, line 5 – remove "novel"

Removed as suggested.

Page 5, line 8 – add space in "damage[2]."

Amended as suggested.

Page 5, line 14 – add a number of participants based on recent reports and data: "13% in select community-based cohorts with laboratory-confirmed COVID-19, to upwards of 71% in hospitalized patients". These data are based only in the UK so we would need to know how many are 13% and 71%?

We thank R2 for their comment but this sentence was used purely to indicate there is a large range in prevalence. As prevalence is typically expressed as a %, we do think reporting the number of participants would add to the manuscript here.

Page 5, line 31 – the authors made an excellent point in "They also reported nearly 70% of patients received a diagnosis of Long-COVID. However, their high prevalence rate was likely influenced by their data coming from a specific post-COVID outpatient service, with most patients having been both hospitalized and intubated during their acute infection. Consequently, it is unclear how well this kind of prospective data reflects the natural progression of the condition in many people who remained community-dwelling and were never hospitalized during the acute phase." This needs a bit more explanation/consideration. The

discussion around Long COVID, with some authors saying that it does not exist, requires attention. I also recommend the authors provide a definition of Long COVID.

We thank R2 for their comment.

Regarding a definition, the UK's National Institute for Health Care and Excellence (NICE) has categorized the duration of these COVID-19 symptoms into three distinct phases: acute (<4 weeks), subacute (4–12 weeks), and chronic (>12 weeks), with the latter two intervals collectively recognized as 'Long-COVID'.

Regarding the difference between hopsitalized and non-hospitalized, this may be explained by one of R1's comments and addressed in our modified introduction:
"The UK's National Institute for Health Care and Excellence (NICE) has categorized the duration of these COVID-19 symptoms into three distinct phases: acute (<4 weeks), subacute (4–12 weeks), and chronic (>12 weeks), with the latter two intervals collectively recognized as 'Long-COVID'. Prevalence estimates for Long-COVID vary, ranging from 13% in select community-based cohorts with laboratory-confirmed COVID-19, to upwards of 71% in hospitalized patients [5–7]. It is worth noting however, that even before COVID-19, nearly one fifth of patients discharged from a hospital develop an acute medical problem within the subsequent 30 days that cause another hospitalization [8], which may account for the range in prevalence rates between hospitalized patients and non-hospitalized patients."

Page 5, line 50 – The authors` aims and objectives are clear. But what is the impact of this research? Why is it important?

We thank R2 for this point. Indeed, it is imperative the importance of our research is outlined. However, we had a significant challenge meeting BMJ's page limits, so have included this information in the discussion as follows:
"This has important repercussions for healthcare practitioners when advising patients on recovery from acute infections, but also economic repercussions, given the large amount of people out of work in the UK as a result of long-term illness [30–32]."

Methods

Page 6, line 30 – please provide details on how "cognitive function" was assessed

We thank R2 for this comment. We have outlined cognitive function assessment as follows:
'Finally, the fourth section of the app contained a Single Digit Modalities Test (SDMT)[24] (LC-COS Cognitive Functioning) for a measure of cognitive function. A series of individual shapes appear on the screen, and the participant must correctly identify which number (0-9) the current shape corresponds to according to the grid at the top of the screen. Participants were scored based on the number of correctly identified shapes and the time to completion.'

Page 6, line 37 – you mentioned that patients need to have a positive COVID test (page 6, line 29), and then you reported that recruiting was completed online. Please clarify if a positive covid test was then self-claimed by the patients or not.

Yes, this was self-reported and we did not query the assay type. The manuscript has now been modified:
"Participants were included in the study if they were adults (≥18 years) living in Scotland and self-report a positive COVID-19 test (any type of assay) within the previous 10 weeks."

Discussion

Page 16, line 10 – the authors discussed long covid in the introduction, then reported fatigue elements in their results and discussion, but failed to link these with their introduction. I recommend talking more about long-term COVID and what others have reported in the literature on this matter. The results on fatigue are extremely valuable and have been well discussed by other authors. My recondition would be to contextualise your results with the current literature knowledge.

We thank R2 for these comments. Again, partially we were limited by page limits so had to reduce the first draft by 40%. However, we agree that the discussion and results did somewhat focus on fatigue (as it was the most prevalent), so it may follow the introduction should do likewise. We have now added some information on fatigue in the second paragraph of the introduction as follows:
"Our recent scoping review highlighted more than 100 symptoms of Long-COVID [9]. However, Davis and colleagues [10] estimated 203 symptoms across 10 organ systems in an online survey of people with suspected and confirmed COVID-19, from 56 countries with a symptom duration of >28 days. This is conflicting with a living systematic review by Michelin at al. [11] who reported over 60 physical and psychological symptoms. It is possible that the study by Michelin et al. [7] may have excluded many studies as an inclusion criterion for individual studies was 'at least 100 people with confirmed or clinically suspected COVID-19 at 12 weeks or more post onset'. In the early phase of the pandemic, most studies concerning Long-COVID had small sample sizes [9] so may have not been included in this systematic review. Regardless of the precise number of symptoms, it is evident from the scientific literature and patient support groups that Long-COVID is a complex condition with heterogeneous symptomology [12]. This makes the formation of a precise case definition or risk evaluation challenging, evidenced by the duration which the Long-COVID Core Outcome Set (LC-COS) took to produce [13]. That being said, now the literature base has increased, it is apparent that fatigue is possible the most common symptom reported by people with Long-COVID [14,15], and qualitative studies have detailed how debilitating this is for people [16,17]."

Limitation

Page 17 – I would suggest that the authors consider their sample of 287 patients. Was this number expected? Where do the authors consider to have a bigger sample size? Given the

number of positive COVID-19 patients, it seems quite a small number. Can the authors include their perspectives on these elements in their limitations?

We thank R2 for this comment and indeed 287 does seem a small n. We found it initially strange we could not recruit more participants, but we attribute this to the following: People already with long-COVID have a community (online support groups on X and meta, charities, diagnoses, etc.). However, we attempted to recruit anyone with a positive test, which is not a community. Anyone already with long-COVID was obviously not eligible as we were investigating the effects of an acute infection so the inclusion criteria was a positive test within 10 weeks.

Of course, in Scotland there were hundreds of thousands of adults with a positive test over the period of our study (12-month recruitment), but UWS is a small post-1992 university with no press office. Thus, recruitment proved challenging and unfortunately relied mostly on snowball sampling using social media posts on Meta and X. This was a bit disappointing.

Conclusion

Page 17, line 47 – add space "Long-COVID[37]"

Amended as suggested.

Page 17 – the "future directions" are missing from "Conclusions and future directions"

We have added the following sentence as future directions:

'Future research may wish to identify risk factors that increase Long-COVID propensity, and of course pharmacological interventions to treat Long-COVID.'

Page 17, line 40 – it is not clear the impact of this research and the take-home point. The authors should use their conclusion to i) state that they met their aims and objectives, ii) clarify the take-home point (not only for academics) but also for health staff, and iii) demonstrate that their research is making an impact.

We apologise but on the pdf our page 17 line 40 is the reference 'Huo T, Guo Y, Shenkman E, et al. Assessing the reliability of the short form 12 (SF-12) health survey in adults with mental health conditions: A report from the wellness incentive and navigation (WIN) study. Health and Quality of Life Outcomes. 2018;16. doi: 10.1186/s12955-018-0858-2'.

However, we assume R2 meant the first paragraph of the discussion (typically a reflection on aims/major findings). We have amended this paragraph to closer reflect the aims as follows:
'This study aimed to (1) evaluate the natural history of symptoms post-infection, (2) detect the proportion of people with persistent symptoms, identify common symptoms associated with COVID-19 recovery, and (3) identify associations of gender and age with symptomology recovery. We feel we met these aims, and the main findings were: (1) around a third of people self-reported full recovery within the first month after infection,

rising to three-quarters by three-months, meaning around a quarter of participants met the criteria for Long-COVID set out by NICE (i.e. symptoms persisting beyond 12-weeks [25]). (2) Although fatigue was the most frequent and enduring symptom, more than half of people had no symptoms or recovered relatively quickly. For some, however, recovery can be slow, with one in five still reporting symptoms after six months and one in thirty at nine months. (3) Time since infection was the only predictor of recovery for most outcomes, with males recovering more quickly for fatigue and PEM than females. (3) A majority experienced minimal to no symptoms, a subset recovered slowly, and a small fraction displayed persistent symptoms throughout the study, was consistent across domains. Consequently, the prevalence of long-COVID varied substantially (between 40% to 3% depending on the time frame (3 months vs 9 months post-infection) and symptoms being considered. Taken together, these data suggest recovery from COVID-19 is slower than from other viral infections and our data highlight the importance of considering duration of infection when assessing Long-COVID prevalence. This has repercussions for healthcare practitioners when advising patients on recovery from acute infections, but also economic repercussions, given the large amount of people out of work in the UK as a result of long-term illness [26–28].'

References

I feel the above comment would also help to improve the number of references to your work.

We thank R2 for their comment, and indeed our number has increased by over 12 (possibly more once we attend to R3's comments).

Reviewer: 3

Dr. Magali Perquin, Luxembourg Institute of Health

Comments to the Author:

It is a nice piece of work. However, some clarifications need to be provided. Please check my detailed review in the attached PDF.

We thank R3 for their comments and kind words.

Please find my comments, questions, and suggestions below, which should be addressed to improve the work. They are listed in the order of appearance in the manuscript (page numbers refer to the BMJ Open PDF):

Line 14 p 5 (Introduction): Please add reference(s) on prevalence ranges (13 to 71%) at the end of the paragraph.

We have added these as recommended.

Lines 5 p 6 (Methods) and Line 23 p 21 (Ethics statement): Is it normal to have a discrepancy in the approval number mentioned: 14019 vs 14988?

We apologise for this oversight. We had two numbers because one was the original submission, and one was the amendment. 14988 is the amendment and thus we have kept this number throughout.

Line 19 p6 (Patients involvement):

- The acronym PPI should be defined here, rather than later;

Amended as suggested.

- This Patient Involvement paragraph should be written in a more neutral tone. Explicit mentions of co-authors and their roles should be omitted from this section and instead included in the contributors section.

W have now removed initials JO, and this section now reads as follows:
"Long COVID Scotland were the participant and patient involvement (PPI) partners for the project and became involved prior to the initial proposal. Our PPI lead became part of the project team and liaised with Long COVID Scotland's members regarding the design, the selection of instruments, and useability testing of the mobile tracking app. Our PPI lead advocated for participants during discussions of study management and progression. Following the project's completion and publication of final results, further feedback will be provided to Long COVID Scotland, who will support further dissemination of the findings through their networks."

Line 28 p6 (Inclusion criteria): Could you please be more specific about the type of positive COVID-19 test required? Are any techniques acceptable?

We thank R3 for their query and yes, any technique was acceptable. The manuscript has been amended thusly:
'Participants were included in the study if they were adults (≥18 years) living in Scotland and had a positive COVID-19 test (any type of test) within the previous 10 weeks.'

Line 48 p6 (Protocol for downloading the app): It is unnecessary to repeat "Once downloaded."

We thank R3 for their attention to detail and have amended the section as follows:

'Once downloaded, participants created an account using a personal email address. Then, the app took participants through a set of onboarding screens designed to explain the features of the app.'

Line 21-22 p7 (Figure 1. caption): The acronyms Eq5D and VAS should be defined again (as TraPSS is).

We have defined the acronyms as suggested.

Line 51 p7 (App design): MRC should be defined (Medical Research Council).

We have defined the acronym as suggested.

Line 9-12 p8 (App design): The description of SDMT could be slightly refined for clarity and conciseness.

We would like to thank R1 for their comment and have amended the section as requested. Remind you script now reads as follows.

"Finally, the fourth section contained a Single Digit Modalities Test (SDMT)[28] (LC-COS Cognitive Functioning). Shapes appeared on the screen, and participants attempted to identify which number (0-9) according to the grid at the top of the screen the shape corresponded to. The number of correctly identified shapes and time to completion were analyzed."

Line 27 p8 (Statistical analysis): The presentation of your model is not so clear to me:

Either you mention a typical linear mixed-effects model such as Outcomein =β0+β1·timein +β2·genderi+β3·agei +β4·(timein·genderi)+β5·(timein·agei)+β6·(genderi·agei)+β7·(timein·genderi·agei)+(1|Subjecti)+in, to examine the effects of time, age, and gender on the construct of recovery. Or, you use the given model (Outcomein=b0in+b1−9time*b4−5*gender*age+ϵi) which is not a traditional linear mixed-effects model. This model presents characteristics more typical of a regression model with complex interactions between time, gender, and age. Can you please clarify this part?

We would like to thank R1 for the suggestion and we have clarified the statistical model as requested.

Line 53 p8 (Results): Before the start of results per se, a basic scheme summarizing the different steps of recruitment process with numbers of participants, loss to follow-up if any etc. would be appreciated.

Moreover, it fits with the STROBE Statement request.

We thank R3 for this suggestion. A figure seems superfluous here because there was no experiment *per se* (as one would expect in a CONSORT; randomised to control/intervention, etc.). However, for clarity, we have added the following text where R3 suggested the information:
"Using an online form, 471 people expressed interest in taking part in the study. We then contacted these 471 people via email to provide study information and attempt to schedule an online meeting. Of these 471 emails, five people provided an ineligible email

address so our email was undelivered. Of the 466 valid emails we sent, 13 people were ineligible (six people had an acute infection >10 weeks previously, six people already had Long-COVID, and one was <18 years of age). Of the remaining 453 people, 141 did not respond to our email. Of the remaining 312, either dropped out between our email and the online meeting or did not attend the online meeting. Of the 288 who attended a meeting and were sent the link to enrol, one person had an incompatible device, so 287 participants were enrolled."

Line 53 p8 (Results): Due to the non-probabilistic nature of the sampling method (snowball sampling), calculating the sample size for the study is indeed challenging. However, could you please explain why 287 participants? Are there some approaches and considerations you use to estimate a sample size needed to achieve certain study objectives?

We thank R3 for this comment. Without known central tendencies and variance of symptoms (this was unknown at the conception of our study), we have presented proportions (much like previous reports in other countries). We were initially surprised by our relatively low recruitment rate, but we suggest this is because people already with long-COVID have a community (online support groups on X and meta, charities, diagnoses, etc.). However, we attempted to recruit anyone with a positive test, which is not a community. Anyone already with long-COVID was obviously not eligible as we were investigating the effects of an acute infection so the inclusion criteria was a positive test within 10 weeks. Of course, in Scotland there were hundreds of thousands of adults with a positive test over the period of our study (12-month recruitment), but accessing those people without specific pathways or ubiquitous characteristics was challenging. Thus, unfortunately we relied mostly on snowball sampling using social media posts on Meta and X. This was a bit disappointing and we would have liked to have included more participants.

Line 6 p9 (Infection recovery and change to work): Figure 1 mentioned here refers rather to Figure 2. Please correct this.

Amended as suggested.

Line 7-8 p9 (Infection recovery and change to work): Please correct the sentence "The 3.1% not reporting 'completely recovered at month 9, reports are of being 'Mostly recovered' (2.4%) or 'about half recovered' (0.7%)." Do you mean: "The 3.1% not reporting 'completely recovered' at month 9, reported being 'Mostly recovered' (2.4%) or 'about half recovered' (0.7%)."?

We have corrected as follows:
'Of the 3.1% not reporting 'completely recovered' at month 9, reported being 'Mostly recovered' (2.4%) or 'about half recovered' (0.7%).'

Line 34 p9 (Figure 2.): It seems that there is an inconsistency regarding the number of months indicated:

it is stated as “9 months” in the text, figure title, and subsequent graphs, whereas in Figure 2, it is shown as 10 months.

Figure 2 has now been edited to show month 0-9.

Line 12 p10 (Symptom frequency): The sentence “64.2% of participants reported some level of fatigue at baseline.” is already written just above (1st line, 1st paragraph).

We thank R3 for this observation and we have removed the second incidence of this text.

Line 15 p10 (Symptom frequency): An inconsistency seems to exist here: in "(decreasing from 37.9% at baseline)". Isn't it 36.9% at baseline (and not 37.9%) as mentioned in the previous paragraph?

We thank R3 for their observation, and have corrected the second number to 36.9%.

Line 28 p10 (Symptom frequency): Is the sign of the estimate (0.09) correct here?

We thank R3 for their observation, and we confirm that the estimate sig and value are correct.

P11-12 (Figure 3): It would be preferable to have all the graphs on the same page/figure. If this is not possible, please provide a title, caption, and figure number for each page (dissociating A to F from G to N).

We agree with R3 this would be easier to read. However, anything we alter at this stage will probably be modified at typesetting (i.e. BMJ open has two lines of text per page, rather than one in the .docx/.pdf.). We will wait until the proofs come through to make this edit if that is acceptable for R3.

Please correct the typo “msucle pain (B)”.

Corrected as suggested.

Line 15-16 p13 (EQ5D): Please use “Impairments in mobility” and “Impairments in self-care” instead of “mobility” and “self-care” to improve clarity. Same suggestion in Figure 4 sub-titles.

We thank R3 for this correction and have made the edits as suggested.

P13 (Figure 4): Please provide a caption for Figure 4 that explains the meaning of 0, 1, 2, 3, 4, etc. Also, check Graph 4C for 'legend'.

‘Legend’ had been removed from the legend. We apologise for this error. We have added the following caption to figure 4 to explain 0-4:

‘0-4 indicate the least to most severe responses to the EQ5D questions (i.e. 0 = ‘I have no problems …’, 4 = I am unable to…’

Line 7 p14 (Dyspnea, VAS pain, anxiety/depression, and self-efficacy): Please clarify the difference between anxiety/depression as presented here and anxiety and depression measured with the EQ5D, as shown in the previous paragraph.

One was EQ5D and one was PhQ4. We have now used subheadings within the manuscript to attempt to clarify this.

Line 36-37 p14 (Cognitive function): Please revise this sentence “This was similar for total time to correct answers at 29.7% at baseline to 90.4% at month 9.”

We have corrected thusly:

‘The number of incorrectly identified objects on the Symbol Digit Modality test reduced over time from 26.0% getting 1 or more incorrect at baseline to 1.8% at month 9. Those taking a short time (<80 s) to complete the test at baseline was 27.6% and increased to 90.4% at month 9. Correct answers increased from 29.7% at baseline to 90.4% at month 9.’

P15 (Figure 5): Please revise the sub-titles G, H, I and refer to SDMT (for example “SDMT: Number of incorrect answers”).

We hade edited the legend to:
‘Figure 5. PhQ4 Anxiety and Depression (A), Self-Efficacy scores (B), MRC Dyspnea scores (C), Visual Analogue Scale Pain (D), Post-exertional malaise frequency (E) and severity (F), Symbol Digit Modalities Test (SDMT): number incorrect (G), SDMT: Total time to completion (H), and SDMT: total time for correct answers (I), from baseline to 9-months.’

P17 (Strengths and limitations):

Line 27: Please correct the typo “focussed”

Corrected as suggested to one s.

Line 30: Please correct “yrs”

Corrected to ‘years’

Line 29-30: Additional potential bias (other than demographic ones) should be mentioned and discussed, such as self-selection bias, network bias etc., all of which can affect the generalizability of the study findings

We thank R3 for this comment, and have added several other limitations as follows:
‘The primary strength of the present study is the use of validated instruments alongside

symptom counts, at a frequency which enabled monthly tracking. This provided a granular view of recovery trajectories from acute COVID-19 infection. Moreover, we purposefully focused on non-hospitalized individuals who have been less well represented in the COVID-19 recovery literature. There are some limitations that should also be considered. Our reliance on mobile technology and social media for recruitment may explain the lack of older participants, thus these findings may not apply to those over 70 years of age. Secondly, following on from the previous limitation around selection bias, as our inclusion criteria was within 10 weeks of testing positive for COVID-19 (the NICE guidelines on what constituted acute, post-acute, and persistent symptoms were still being developed when this study commenced), 10 weeks falls within the subacute phase, and those people may be more likely to progress into the chronic phase than those who have no symptoms. As such, perhaps those that consider themselves having ongoing symptoms participated, resulting in a selection bias. Thirdly, it is difficult to separate those with Long-COVID (caused by an acute COVID-19 infection) from those with post-vaccine syndrome [45], or to give its colloquial term, 'Long Vax(x)' [46]. Indeed, Arjun et al. [47] noted a greater risk (adjusted odds ratio of 2.32) of Long-COVID symptoms in those with two vaccine doses. As 99% of our participants were vaccinated, it is possible that a proportion of people reporting symptoms at 9 months were because of Long Vaxx as opposed to Long-COVID. Penultimately, our list of symptoms was evidence based [5] and developed with our PPI group (Long-COVID Scotland), presenting participants with a pre-defined list of Long-COVID symptoms may have limited the range or specificity of symptoms reported by participants. Finally, reporting of new infections was low and the possibility of underreporting cannot be discounted. At that time, 1-2% of the Scottish population was testing positive for COVID-19 [48]. If underreporting was present, re-infections may have contributed to persistent or re-occurring symptoms.'

Instructions:

We now have three reviews on your work - please see below and the attached pdf - and I hope you find the comments constructive. We would like to invite you to revise the paper in response to the reviews, and the editors' comments (below my signoff) - please understand your revision may be subject to further review and that this initial decision does not guarantee acceptance.

Please read and respond to all of the peer review and editorial comments. You should provide a point-by-point response to explain any changes you have (or have not) made to the original article and be as specific as possible in your responses.

The original files will be available to you when you start your revision. Please delete any files that you intend to replace with updated versions and upload the following using the appropriate file designation:

- “Main Document’’ - This is a clean copy (without tracked or highlighted changes) of your revised article. Please delete your original submission file.

- “Main Document - marked copy” - This is the edited version of your original article, including edits to address the peer review comments. Any changes have been highlighted using a track change function or bold or coloured text.

Please replace any other files that have been updated e.g. Images, forms.

The reviewers' comments, your response, and the previous versions of your article will be published as supplementary information alongside the final version of your article.

Information relating to your article, including author names and affiliations, title, abstract and required statements (e.g. competing interests, contributorship, funding) will be taken directly from the information held in ScholarOne, and not from the article file. Please check that this information has been entered correctly and has been updated as appropriate. If your revised article is accepted, you will only be able to make minor changes (e.g. correction of typesetting errors and proof stage) prior to publication.

VERSION 2 - REVIEW

**Reviewer**	**4**
**Name**	**Kelson, Zoe**
**Affiliation**	**University of Exeter, Mathematics**
**Date**	**28-Nov-2024**
**COI**	

The aim of this project was to track symptoms of individuals following a COVID-19 infection for 9-months using a specially designed mobile health app to determine symptomology changes over time and to undertake regular assessments with validated instruments.

Reviewer comments:

"Fatigue, cough, and muscle pain were the most common symptoms at baseline, with fatigue persisting the longest, while symptoms like cough improved rapidly. Older age increased the likelihood of reporting pain and cognitive impairment. Female gender increased the likelihood of headaches and post-exertional malaise (PEM), and increased recovery time from fatigue and PEM. "

Can statistical outcomes please be reported in the Abstract?

"self-report a positive COVID-19 test (any type of assay) within the previous 10 weeks"

Can the authors please discuss the potential for self-reporting bias?

"Respondents were excluded for insufficient English language for messaging to be effective; no mobile device access; impaired cognitive function which compromized comprehension of study information or messaging; current participation in any COVID-19 intervention, receiving therapies known to cause symptom exacerbations (e.g. chemotherapy)."

Can language-based selection bias please be discussed?

"Participants were recruited via snowball sampling using social media posts on Facebook/Meta and Twitter/X between January 2022 and January 2023."

Can the authors please also discuss possible sampling bias (i.e. not a random sample)?

"To examine the effects of time, age, and gender on the construct of recovery, we used the following linear mixed-effects model...

All domains of recovery represented the repeated-measures outcome for subjectin and served as outcome measures whereas time (continuous variable with 9 levels [consecutive months]), gender (categorical variable with 2 levels [female and male]) and age (continuous variable) were modelled as predictors and treated as fixed effects alongside their three-way and two-way pairwise interactions. Moreover, random effects were assumed for participants, with random slopes per the predictor time introduced in the model as this addition did not result in a convergence error."

and

"Estimated marginal means and 95% confidence intervals were calculated alongside comparisons made using post-hoc Holm-Bonferroni adjustments. Visual inspection of residual plots was used to confirm the assumptions of homoscedasticity or normality, which was also assessed through the Shapiro-Wilk test. Moreover, since regression models can be sensitive to multicollinearity, we computed the variance inflation factors for all predictor parameters used in the linear mixed-effects model to inspect the presence of autocorrelation between pairs of predictors. Model residuals were qualitatively examined for structure and heteroscedasticity. We computed 90% CIs of the adjusted effects using the bias-corrected and accelerated bootstrap with 5,000 replicates."

Appropriate modelling methods have been applied by the authors.

"We assumed that data were missing at random"

Can the authors please clarify how much data was missing, and how this was handled in the analysis?

"so 287 participants were enrolled... Participant demographics can be found in supplementary table 1."

Can the authors please comment on whether the included sample can be considered to be demographically and characteristically representative?

The results are presented accurately, and the figures are informative.

"A limitation is that at the time of study commencement, definitions around acute, post-acute, and persistent symptoms were still being refined by NICE. • A second limitation is the moderate sample size."

and

"There are some limitations that should also be considered. Our reliance on mobile technology and social media for recruitment may explain the lack of older participants, thus these findings may not apply to those over 70 years of age. Secondly, following on from the previous limitation around selection bias, as our inclusion criteria was within 10 weeks of testing positive for COVID-19 (the NICE guidelines on what constituted acute, post-acute, and persistent symptoms were still being developed when this study commenced), 10 weeks falls within the subacute phase, and those people may be more likely to progress into the chronic phase than those who have no symptoms. As such, perhaps those that consider themselves having ongoing symptoms participated, resulting in a selection bias. Thirdly, it is difficult to separate those with Long-COVID (caused by an acute COVID-19 infection) from those with post-vaccine syndrome [53], or to give its colloquial term, 'Long Vax(x)' [54]... Finally, reporting of new infections was low and the possibility of underreporting cannot be discounted. At that time, 1-2% of the Scottish population was testing positive for COVID-19 [56]. If underreporting was present, re-infections may have contributed to persistent or re-occurring symptoms"

Can the limitations acknowledged in the 'strengths and limitations of this study' and discussion sections please be expanded to further address representativeness (e.g. the lack of a random sample and ethnic diversity), as well as the sample size in the discussion?

Thanks for providing a copy of the STROBE checklist.

VERSION 2 - AUTHOR RESPONSE

Reviewer4 comments:

1. **"Fatigue, cough, and muscle pain were the most common symptoms at baseline, with fatigue persisting the longest, while symptoms like cough improved rapidly. Older age increased the likelihood of reporting pain and cognitive impairment. Female gender increased the likelihood of headaches and post-exertional malaise (PEM), and increased recovery time from fatigue and PEM. "**
   Can statistical outcomes please be reported in the Abstract?

We thank R4 for their comment and have now included statistical outcomes in the abstract thusly: "**Results:** 3.1% of participants experienced symptoms at month 9, meeting the criteria for Long-COVID, as defined by the NICE terminology. The random effects model revealed a significant time (month) effect for infection recovery ($p<0.001$, estimate=0.07). Fatigue, cough, and muscle pain were the most common symptoms at baseline, with fatigue persisting the longest, while symptoms like cough improved rapidly. Older age increased the likelihood of reporting pain ($p=0.028$, estimate=0.07) and cognitive impairment ($p<0.001$, estimate=0.93). Female gender increased the likelihood of headaches ($p=0.024$, estimate=0.53) and post-exertional malaise (PEM) frequency

(p=0.05, estimate=137.68), and increased time x gender effect for PEM frequency (p=0.033, estimate=18.96).”

2. **"self-report a positive COVID-19 test (any type of assay) within the previous 10 weeks". Can the authors please discuss the potential for self-reporting bias?**

Thank you for highlighting this bias which we previously omitted from the limitations section. We have now included within the “Strengths and Limitations” subheading of the Discussion and acknowledgement that self-report bias may have led to participants with symptoms but no positive test signing on to the study. The text is as follows:

“Those that consider themselves having ongoing symptoms participated, resulting in a selection bias, and those with on-going symptoms but no positive test may have signed up to take part resulting in self-report bias. Indeed, the use of snowball sampling may have biased the sample further as those with prolonged symptoms highlighted the study specifically to others with similar conditions.”

3. **"Respondents were excluded for insufficient English language for messaging to be effective; no mobile device access; impaired cognitive function which compromized comprehension of study information or messaging; current participation in any COVID-19 intervention, receiving therapies known to cause symptom exacerbations (e.g. chemotherapy)." Can language-based selection bias please be discussed?**

Thank you for raising this important point. We have now included this section to the discussion section to acknowledge this study limitation:

“Thirdly, excluding individuals with insufficient English proficiency may further limit generalisability, as these individuals may differ in health-seeking behaviours, resource access, or cultural attitudes. This exclusion could disproportionately affect non-native speakers, overlapping with underserved groups and potentially perpetuating health disparities.”

4. **"Participants were recruited via snowball sampling using social media posts on Facebook/Meta and Twitter/X between January 2022 and January 2023." Can the authors please also discuss possible sampling bias (i.e. not a random sample)?**

Thank you for highlighting this bias which we previously omitted from the limitations section. We have now included a brief discussion within the “Strengths and Limitations” subheading of the Discussion. Noting that snowball sampling may have led to targeted sampling on social media as those with prolonged symptoms highlight the study to others with similar conditions. We found recruitment challenging as our criteria was to include anyone with an acute infection and these people do not fall into a specific category or group of people. In our **Long**-COVID work, our ME/CFS work, our MS work, and our ageing work, there are specific online groups and charities which make recruitment easier. However, for acute infections, this was essentially the entire population, and although this may seem easier to recruit (as there are more people), we observed the opposite, because the criteria was so broad, there were no bespoke groups for acute infection ‘sufferers’.

5. **"To examine the effects of time, age, and gender on the construct of recovery, we used the following linear mixed-effects model...**
   All domains of recovery represented the repeated-measures outcome for subjectin and served as outcome measures whereas time (continuous variable with 9 levels [consecutive months]), gender (categorical variable with 2 levels [female and male]) and age (continuous variable) were modelled as predictors and treated as fixed effects alongside their three-way and two-way pairwise interactions. Moreover, random effects were assumed for participants, with random slopes per the predictor time introduced in the model as this addition did not result in a convergence error."
   and

"Estimated marginal means and 95% confidence intervals were calculated alongside comparisons made using post-hoc Holm-Bonferroni adjustments. Visual inspection of residual plots was used to confirm the assumptions of homoscedasticity or normality, which was also assessed through the Shapiro-Wilk test. Moreover, since regression models can be sensitive to multicollinearity, we computed the variance inflation factors for all predictor parameters used in the linear mixed-effects model to inspect the presence of autocorrelation between pairs of predictors. Model residuals were qualitatively examined for structure and heteroscedasticity. We computed 90% CIs of the adjusted effects using the bias-corrected and accelerated bootstrap with 5,000 replicates."

Appropriate modelling methods have been applied by the authors.

We thank R4 for their positive comment on our analysis.

6. **"We assumed that data were missing at random". Can the authors please clarify how much data was missing, and how this was handled in the analysis?**

We thank R4 for their comment. Importantly, our linear mixed-effects statistical models do not require data imputation so there was no handling of missing data. We have clarified this in the methods as follows:

"We assumed data were missing at random and linear mixed effects models handle missing data without requiring imputation [33].

7. **"so 287 participants were enrolled... Participant demographics can be found in supplementary table 1." Can the authors please comment on whether the included sample can be considered to be demographically and characteristically representative?**

We thank R4 for their comment. According to the most recent census data, our sample were representative in terms of average age of the Scottish population. However, concerning gender (we asked participants for their gender, not sex), 51% of the population are female according to the census, but 78% of our sample was female. We have acknowledged this in the limitations as follows:

"Our sample demographics speak to this, as 78% of our sample was female but only 51% of the Scottish population is female and long COVID is known to disproportionately affect females [18,55]."

8. **The results are presented accurately, and the figures are informative.**

We thank R4 for their positive comment regarding our data visualisation.

9. **"A limitation is that at the time of study commencement, definitions around acute, post-acute, and persistent symptoms were still being refined by NICE. A second limitation is the moderate sample size."**

and

"There are some limitations that should also be considered. Our reliance on mobile technology and social media for recruitment may explain the lack of older participants, thus these findings may not apply to those over 70 years of age. Secondly, following on from the previous limitation around selection bias, as our inclusion criteria was within 10 weeks of testing positive for COVID-19 (the NICE guidelines on what constituted acute, post-acute, and persistent symptoms were still being developed when this study

commenced), 10 weeks falls within the subacute phase, and those people may be more likely to progress into the chronic phase than those who have no symptoms. As such, perhaps those that consider themselves having ongoing symptoms participated, resulting in a selection bias. Thirdly, it is difficult to separate those with Long-COVID (caused by an acute COVID-19 infection) from those with post-vaccine syndrome [53], or to give its colloquial term, 'Long Vax(x)' [54]... Finally, reporting of new infections was low and the possibility of underreporting cannot be discounted. At that time, 1-2% of the Scottish population was testing positive for COVID-19 [56]. If underreporting was present, re-infections may have contributed to persistent or re-occurring symptoms"

Can the limitations acknowledged in the 'strengths and limitations of this study' and discussion sections please be expanded to further address representativeness (e.g. the lack of a random sample and ethnic diversity), as well as the sample size in the discussion?

We thank the reviewer for this comment and have cited representativeness (age/gender) in the limitations and increased this section in line with editor and reviewer comments: "Our reliance on mobile technology and social media for recruitment may explain the lack of older participants, thus findings herein may not apply to those over 70 years of age. This is a distinct limitation given the impact of acute COVID-19 infection on the over 70s in the knowledge that 14% of the Scottish population are over 70 years of age [54]. Secondly, our inclusion criteria was within 10 weeks of a self-reported positive test for COVID-19 (the NICE guidelines on what constituted acute, post-acute, and persistent symptoms were still being developed when this study commenced), 10 weeks falls within the subacute phase, and those people may be more likely to progress into the chronic phase than those who have no symptoms. As such, two potential sampling biases may have occured. Those that consider themselves having ongoing symptoms participated, resulting in a selection bias, and those with on-going symptoms but no positive test may have signed up to take part resulting in self-report bias. Our sample demographics speak to this, as 78% of our sample was female but only 51% of the Scottish population is female and long COVID is known to disproportionately affect females [18,55]. Indeed, the use of snowball sampling may have biased the sample further as those with prolonged symptoms highlighted the study specifically to others with similar conditions. Thirdly, excluding individuals with insufficient English proficiency may further limit generalisability, as these individuals may differ in health-seeking behaviours, resource access, or cultural attitudes. This exclusion could disproportionately affect non-native speakers, overlapping with underserved groups and potentially perpetuating health disparities. That being said, in the last census 98.6% of people in Scotland aged 3 and over spoke English [54]"

Regarding sample size, we have given a detailed response to editor comment 3 above. In summary outcomes of a statistical power simulation based on the final sample size and our study design, with a Type-1 error rate of 0.05, a total sample size of 287 subjects from two subgroups (males and females), 5-7 measurements (monthly reports), and a conservative correlation among repeated measures of 0.5, the statistical power to detect a significant effect was calculated at 0.88-0.96 for all outcome measures.

Thanks for providing a copy of the STROBE checklist.
